# Prep1 prevents premature adipogenesis of mesenchymal progenitors

**DOI:** 10.1038/s41598-017-15828-1

**Published:** 2017-11-14

**Authors:** Giorgia Maroni, Vsevolod A. Tkachuk, Alexander Egorov, Marco J. Morelli, Raffaele Luongo, Elena Levantini, Francesco Blasi, Maria Cristina Magli, Dmitry Penkov

**Affiliations:** 1 Institute of Biomedical Technologies, National Research Council (CNR), Via Moruzzi, 1, 56124 Pisa, Italy; 2 Lomonosov Moscow State University, Leninskiye Gory 1, 119991 Moscow, Russia; 3 Center for Genomic Science of IIT@SEMM, Fondazione Istituto Italiano di Tecnologia (IIT), via Adamello 16, 20139 Milano, Italy; 4 Beth Israel Deaconess Medical Center, 330 Blackfan Circle, CLS 428, Boston, 02215 MA USA; 5 IFOM (The FIRC Institute of Molecular Oncology), via Adamello 16, 20139 Milano, Italy; 6 National Medical Research Center for Cardiology, 3rd Cherepkovskaya Street 15A, 121552 Moscow, Russia; 7 Present Address: Beth Israel Deaconess Medical Center, 330 Blackfan Circle, CLS 428, Boston, 02215 MA USA; 8 Present Address: European Institute of Oncology, via Adamello 16, 20139 Milano, Italy

## Abstract

Transcriptional regulators are crucial in adipocyte differentiation. We now show that the homeodomain-containing transcription factor Prep1 is a repressor of adipogenic differentiation since its down-regulation (DR) in both *ex vivo* bone marrow-derived mesenchymal stromal cells (MSC) and *in vitro* 3T3-L1 preadipocytes significantly increases their adipogenic differentiation ability. Prep1 acts at a stage preceding the activation of the differentiation machinery because its DR makes cells more prone to adipogenic differentiation even in the absence of the adipogenic inducers. Prep1 DR expands the DNA binding landscape of C/EBPβ (CCAAT enhancer binding protein β) without affecting its expression or activation. The data indicate that Prep1 normally acts by restricting DNA binding of transcription factors to adipogenic enhancers, in particular C/EBPβ.

## Introduction

Adipogenesis mostly takes place during embryonic development and childhood (in the mouse it starts immediately after birth and proceeds during the immediate postnatal days), however, it also occurs in adult life, when 10% of fat cells annually renew^1^. Adipogenesis can also be abnormally activated throughout life in obese individuals^2^, and the mechanisms leading to such dysregulation are not yet totally understood. Mature adipocytes derive from not yet well characterized mesenchymal stem (stromal) cells (MSC)^3^, however several proteins that regulate adipogenesis at different stages of differentiation have been identified mainly via *in vitro* experiments. In this respect, activation of hormone signaling pathways and chromatin remodeling that occur within hours from induction are critically important^4–6^. Adipogenic differentiation can be stimulated *in vitro* by different soluble inducers^7^. In the preadipocyte cell line 3T3-L1, frequently used as a model to study the adipogenic process^8^, the addition of insulin, dexamethasone and 3-isobutyl-1-methylxanthine (IBMX), hence referred to as the complete adipogenic differentiation cocktail, leads to the very early recruitment of transcription factors into well defined chromatin hotspots hosting multiple transcription factor binding sites characterized as adipogenic enhancers^9^. These hotspots are always occupied by C/EBPβ, which appears to be the most-upstream triggering transcription factor, whereas Pparγ (Peroxisome proliferator-activated receptor gamma), one of the main players in the adipogenic lineage, seems to act downstream^10^. There is evidence indicating that also the homeoprotein Pbx1 is an important transcription factor in adipogenic differentiation as its down-regulation (DR) activates adipogenesis^11^. It is known that another homeodomain transcription factor of the TALE class, Prep1, is an essential partner of Pbx1^12^, and we herein investigated its role in adipogenic differentiation.

Prep1 is essential in embryonic development. *Prep1* ^−/−^ embryos die very early at the epiblast stage without reaching gastrulation^13^ whereas hypomorphic *Prep1* ^*i*/*i *^embryos, that express around 2% of normal Prep1 mRNA, have a milder (embryonic lethality at E17.5), not always penetrant, phenotype^14^. Foetal liver *Prep1* ^*i*/*i *^cells lack active hematopoietic stem cells and are unable to repopulate irradiated hosts^15^.

Prep1 reaches the nucleus and binds DNA only as a dimer with Pbx1^16,17^. Indeed, Prep1 cooperates with Pbx1 to regulate *Hox* gene expression^12,14,18^. We recently described the Prep1 and Pbx1 binding sites to the DNA of mouse embryos, embryonic stem cells and embryonic fibroblasts and showed that Prep1 mostly, but not exclusively, binds DNA as a dimer with Pbx1 and that two thirds of the binding sites are cell-type specific^12,19–21^.

Prep1 is also involved in cell differentiation as indicated by the inability of *Prep1* ^*i*/*i*^ B and T cells to properly differentiate^14,22^, although the mechanisms are still unknown.

In this paper we report that in both *ex vivo* bone-marrow derived MSCs and in *in vitro* 3T3-L1 cells, Prep1 DR, similar to the effect of Pbx1 DR^11^, results in activation of adipogenesis. Interestingly, Prep1 DR triggers adipogenic differentiation even in the absence of complete hormonal induction, since Prep1 down regulated cells are already poised towards adipogenesis before the addition of the inducing hormones. Prep1 DR results in the expansion of the C/EBPβ DNA binding landscape already before induction and its ability to bind DNA is increased after the addition of the adipogenic inducers. Prep1 does not control a specific “differentiating” mechanism, but rather predisposes the cells towards adipogenic differentiation; therefore its possible role appears to be that of restricting DNA binding of transcription factors, in particular C/EBPβ, for example as a chromatin remodeling factor.

## Results

### Prep1 Down Regulation stimulates adipogenic differentiation

**Figure 1:**
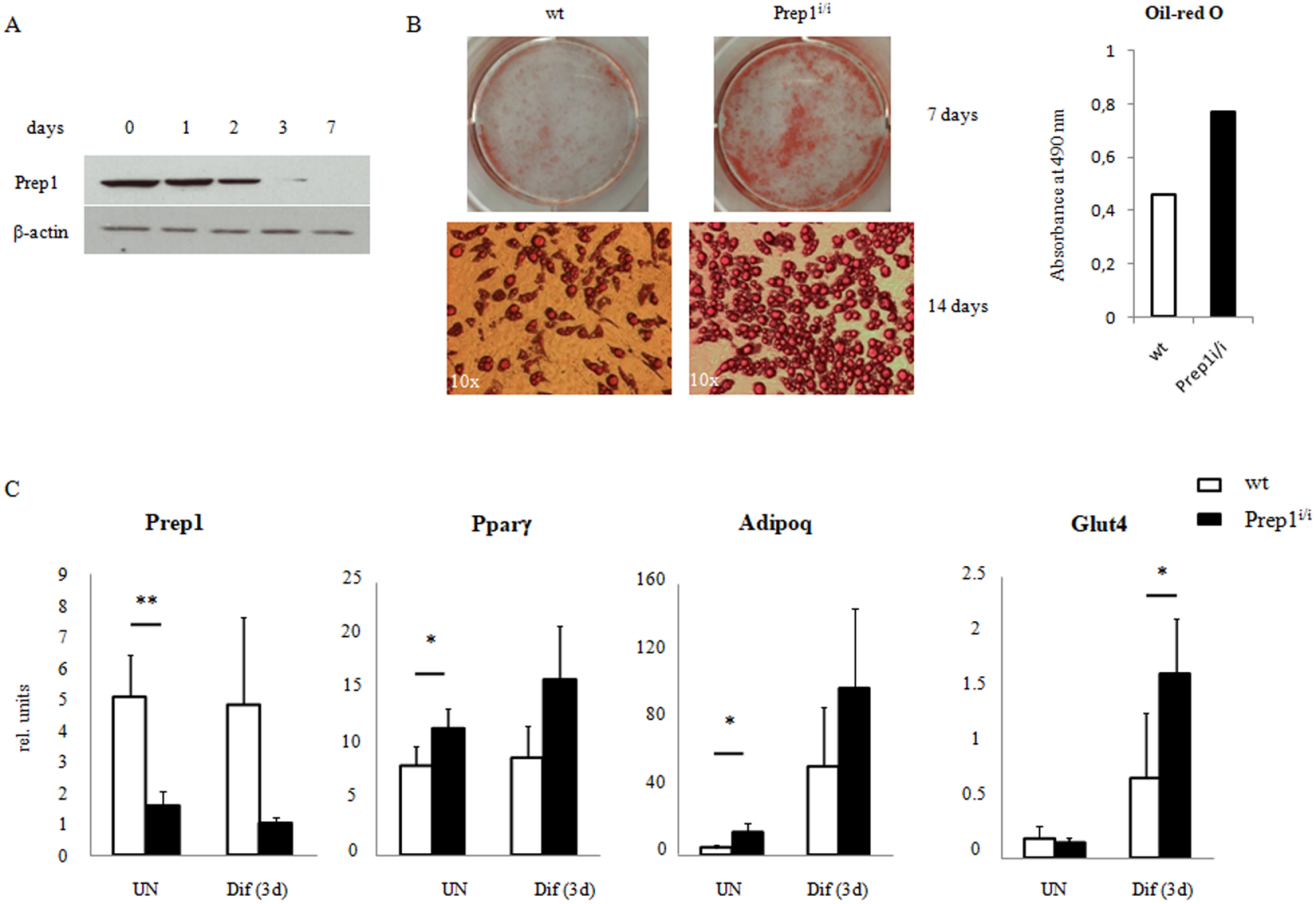
Bone marrow derived Mesenchymal Stromal Cells (MSC) from Prep1 hypomorphic mice exhibit enhanced adipogenic differentiation potential. (**A**) Immunobloting analysis of Prep1 protein in MSCs at different times of adipogenic differentiation. Days after the induction of differentiation are shown. β-actin Ab was used as a loading control. Full-length blots are presented in Supplementary Fig. S1. (**B**) Oil-red O staining of MSCs from wild-type (wt) or Prep1 hypomorphic (*Prep1* ^*i/i*^) mice bone marrow 7 or 14 days after addition of the differentiation cocktail. Quantification of Oil-red O staining by absorbance at 490 nm is shown. (**C**) qRT-PCR analysis of *Prep1*, *Ppar*γ, *Adipoq* and *Glut4* expression in MSCs from wild-type (wt) or Prep1 hypomorphic (*Prep1* ^*i*/*i*^) mice before (uninduced, UN) and 3 days after (Dif (3d)) induction of adipogenic differentiation. Each graph is representative of 3 independent experiments. Level of significance is indicated as follows *p ≤ 0.05, **p ≤ 0.01.

As a first step to test the role of Prep1 in adipogenic differentiation we used *ex vivo* bone marrow-derived MSCs. Initially, we analyzed *Prep1* expression, both at the transcriptional and protein levels, in undifferentiated MSCs and at different time points following adipogenic induction, using MesenCult™ Adipogenic Stimulatory Supplement (Stem Cell Technologies). Prep1 protein is present in wild type MSCs, but its expression level rapidly decreases after 3 days of differentiation (Fig. 1A) while its RNA level remains unchanged (Fig. 1C). We investigated the effects of Prep1 DR *in vivo* by using a hypomorphic murine line in which the levels of Prep1 protein are 2–20% of the wt levels^14^. Therefore, we set up MSC cultures, from both *Prep1* ^*i*/*i*^ and wt mice, studied their growth and differentiation ability, following adipogenic induction. Undifferentiated *Prep1* ^*i*/*i*^ and wt cells, collected from n = 8 mice per genotype, did not show differences in terms of morphology and growth kinetics in culture. In contrast, upon adipogenic induction, we observed in 4 out of 8 bone marrow samples that *Prep1* ^*i*/*i*^ MSCs differentiated much faster than *Prep1* ^+/+ ^control cells, as evidenced by a faster lipid droplet accumulation. Seven days after induction the adipogenic differentiation of *Prep1* ^*i*/*i*^ cells was almost complete, while much less *Prep1* ^+/+ ^control cells contained lipid droplets (mature adipocytes) (Fig. 1B). Quantification of lipids by Oil-red O staining at terminal differentiation (14 days) showed a two-fold (1.67-fold) higher level in *Prep1* ^*i*/*i *^than in control cells (Fig. 1B). Both the observation that *Prep1* expression decreases during wt adipogenic differentiation and the data showing effects of Prep1 *ex vivo* DR suggest that Prep1 plays a negative regulatory role in the adipogenic program. In addition, we correlated the expression of *Prep1* and selected markers of adipogenic differentiation in wt and mutant MSCs, before and after adipogenic induction (3 days). As expected, we detected a significant reduction of *Prep1* expression in undifferentiated *Prep1* ^*i/i*^ MSCs, as compared to wt cells (Fig. 1C). Moreover, Prep1 RNA levels of both wt and mutant cells remain virtually unchanged between undifferentiated and early differentiated cells. Consistently, we observed that the expression of critical mediators of adipogenesis is higher in *Prep1* ^*i*/*i *^cells, already at early time points after adipogenic induction (Fig. 1C). Interestingly, the mRNA levels of the early adipogenic markers Pparγ (p = 0.035) and adiponectin (Adipoq; p = 0.05) are significantly increased in *Prep1* ^*i*/*i *^MSCs, even in the absence of adipogenic induction, suggesting that Prep1 DR itself favors differentiation of MSCs towards an adipogenic fate. All the data obtained indicate that indeed *Prep1* ^*i/i*^ MSCs differ from their normal counterparts already at the undifferentiated state.

We observed these results only in MSC cells derived from 50% of the *Prep1* ^*i*/*i*^ bone marrows (n = 8). This is in perfect agreement with the non-penetrant embryonic and adult phenotype of these mice, as only 75% of the embryos die *in utero* and only 50% of the adult *Prep1* ^*i*/*i*^ mice develop tumors^14,23^.

**Figure 2:**
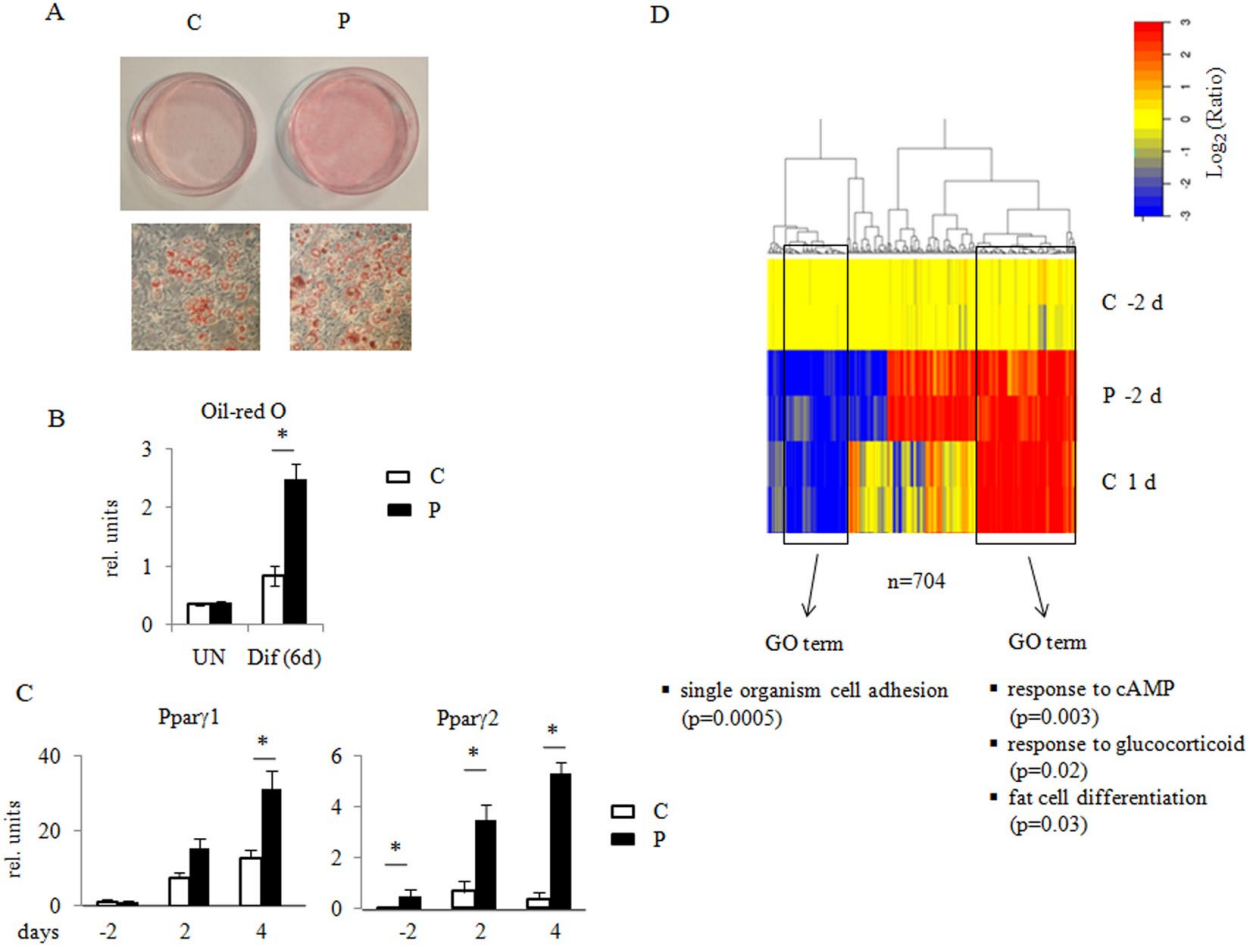
Prep1 down-regulation in 3T3-L1 cells results in an increased tendency towards adipogenic differentiation. (**A**) Oil-red O staining of 3T3-L1 control (C) and Prep1 down-regulated (P) cells 6 days after induction of adipogenic differentiation. (**B**) Quantification of Oil-red O staining by absorbance at 490 nm. UN - undifferentiated (n = 4); Dif (6d) - after 6 days of differentiation (n = 4), *p < 0.01. (**C**) qRT-PCR analysis of Pparγ1 and Pparγ2 RNA transcripts in 3T3-L1 cells before (−2), 2 (2) and 4 days (4) after adipogenic differentiation. Each graph is representative of 3 independent experiments +/− std. deviation. *p < 0.01. (**D**) Clustering heatmaps of gene expression changes assayed by RNA-seq from P and C cells before differentiation (−2 days) and control cells 1 day after differentiation (1 day), where n = 704 represents the number of the genes that changed their expression. The logarithm of ratios for each normalized RPKM is shown. Gene ontology (GO) terms of 3T3-L1 genes enriched by both differentiation of control cells (from day −2 to day +1) and by Prep1 down-regulation at day −2 are shown.

Given the above observations obtained in *Prep1* ^*i/i*^ mesenchymal stromal cells and the intrinsic high levels of heterogeneity of MSCs, we decided to adopt a more homogeneous cell system to further investigate Prep1 function in the adipogenic process. In particular, we adopted the 3T3-L1 cell line frequently used as a model of *in vit* *ro* adipogenesis, and infected it with a lentivirus vector carrying a scrambled control virus (henceforth referred to as C cells) or two different Prep1 shRNAs (named P and P2 cells, respectively) (Supplementary Fig. S2A). We verified that *Prep1* expression level is maintained at a down-regulated status at least for three days after induction (Supplementary Fig. S2B). Figure 2A,B show that six days after induction, P cells presented at least twice more lipid droplets than C cells (p < 0.01). Similar results were obtained using the second shRNA2 (Supplementary Fig. S2C). The mRNA levels of the early adipogenic markers Pparγ1 and Pparγ2 are significantly increased (Fig. 2C). Therefore, a negative effect of Prep1 on adipogenesis was evident from both *ex vivo* derived MSCs and the already committed preadipocyte cell line 3T3-L1.

### Prep1 DR activates core set of adipogenic genes at a pre-induction stage

In order to explore at the transcriptional level the mechanism leading to the enhanced differentiation of P cells, we performed RNA-seq in C and P cells before (day −2) and 24 hours after induction of differentiation (accessible in GEO at the accession number GSE100057). An expression ratio >2 or <0.5 was used as threshold to identify up- and down-regulated genes, respectively. In C cells, the adipogenic-inducing cocktail affected the expression of 2,800 genes after the first 24 hours of differentiation (Table 1). In fact, in P cells the total number of affected genes was lower because ~700 genes were already up- (446 genes) or down- regulated (259 genes) with respect to C cells before the induction (Table 1).

**Table 1:** RNA-seq analysis of the effect of Prep1 DR on differentiated and undifferentiated 3T3-L1 cells^#^.

	Effect of Differentiation in C cells* Number of genes.	Effect of Differentiation in P cells* Number of genes.	Comparison of P v. C cells before differentiation^$^ Number of genes.	Comparison of P v. C cells 24 h after induction^$^ Number of genes.
Up-regulated	1731	896	446	192
Down-regulated	1069	1077	259	346

^#^The data refer to the number genes up- (>2x) or down-regulated (>0.5x) as obtained by the RNA-seq data (padj < 0.1).

*Differentiated C or P cells 24 hrs after the addition of the inducing cocktail are compared with non differentiated cells (two days before the addition of the inducing cocktail).

^$^Comparison between C and P cells 2 days before or 24 h after the addition of the inducing cocktail.

A heatmap with hierarchical clustering (Fig. 2D) showed the ratios between the RPKM (reads per kb per million aligned reads) of each gene versus the average of two control replicas at time −2 days. We included only those genes whose expression ratio was >2 or <0.5 and Benjamini-Hochberg adjusted p-value < 0.1. This analysis showed that a subset of the genes regulated by the inducing hormones in C cells, is already affected in the same direction in P cells before differentiation, i.e. in the absence of the hormones. This strongly suggests that endogenous Prep1 normally prevents adipogenic differentiation by some mechanism that can be counteracted by the inducing hormones.

The list of genes that are similarly affected by Prep1 DR at day −2 and by induction of adipogenic differentiation of control cells at day 1, is shown in Supplementary Table S1. Gene Ontology (GO) shows a statistically significant enrichment for the gene categories highlighted in Fig. 2D and listed in Supplementary Table S2. The genes up-regulated in P cells belong to GO categories such as response to cAMP (p < 0.0004), response to glucocorticoid (p < 0.005), fat cell differentiation (p < 0.02). These data are in agreement with the observed phenotype displaying higher adipogenesis.

Overall, these results show that in 3T3-L1 cells Prep1 DR induces a major alteration in the gene expression pattern even before the adipogenic induction confirming the hypothesis that Prep1 acts as an inhibitor for the adipogenic differentiation program.

### Prep1 DR affects pathways induced by all three adipogenic inducers

**Figure 3:**
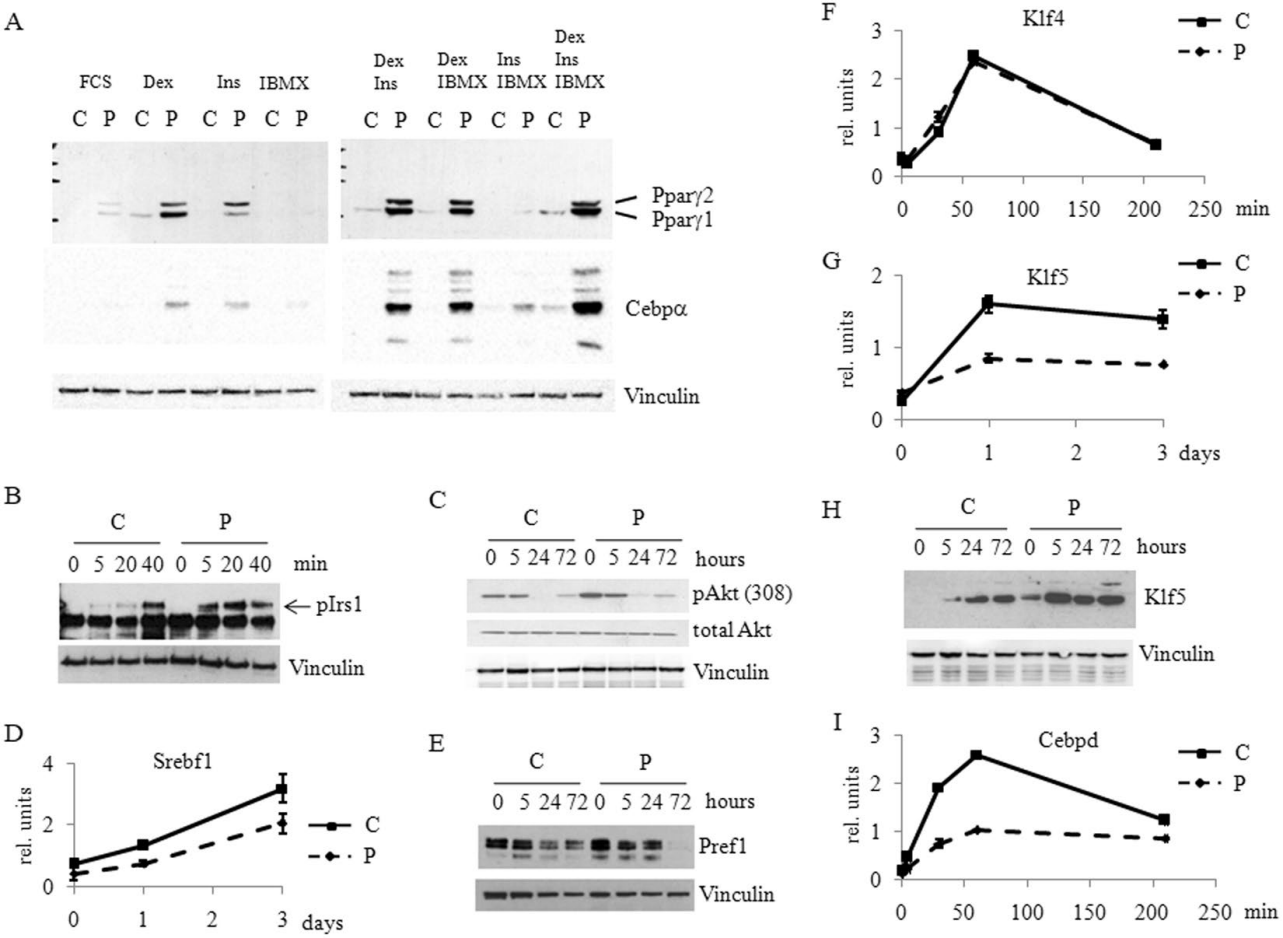
Prep1 down-regulation affects the expression and phosphorylation of key genes and proteins involved in adipogenic differentiation. (**A**) Immunoblotting analysis of Pparγ, C/EBPα and Vinculin (for loading control) in total cell lysates from C and P cells 3 days after induction of differentiation. For the induction Ins, Dex and IBMX as single agents in the presence of FCS or in various combinations, as indicated, were used. Full-length blots are presented in Supplementary Fig. S3. (**B**) Immunoblotting analysis of cell extracts from Prep1 down-regulated (P) and control cells (C) at various time points after induction using anti-phospho-Irs1 (Tyr941) Ab. Dex, Ins and IBMX in the presence of FCS were used to induce differentiation. The arrow shows the size of the phosphorylated form (pIrs1). Vinculin Ab was used to assess loading. Full-length blots are presented in Supplementary Fig. S4. (**C**) Immunoblotting of cell extracts from P and C cells at various time points after differentiation induction using anti-phospho-Akt (Thr308) Ab. Total Akt and Vinculin Abs were used as controls. Full-length blots are presented in Supplementary Fig. S5. qRT-PCR analyses of *Srebf1* (**D**), *Klf4* (**F**), *Klf5* (**G**) and *Cebpd* (**I**) expression in P (solid line) and C (dashed line) cells at different time points after induction. Relative gene expression was normalized to the levels of *Gapdh* expression. Immunoblotting of cell extracts from P and C cells at various time points after differentiation induction using anti-Pref1 (**E**) and anti-Klf5 (**H**) Abs. Vinculin Ab was used as loading control. Full-length blots are presented in Supplementary Figs S7 and S8, respectively.

Given the observed changes in gene expression following Prep1 DR, one would expect that Prep1 may not specifically affect the action of a single adipogenic inducer. Therefore, to test such hypothesis, cells were incubated in media containing only one of the hormones needed for differentiation, i.e. insulin (Ins), dexamethasone (Dex) or IMBX^24^ starting two days after reaching confluence and the expression of key adipogenic markers was followed for 3 days. Pparγ1 and Pparγ2, two important mediators of adipogenesis, were induced more strongly in P rather than C cells also in the presence of Ins or Dex only. The same was observed for C/EBPα (Fig. 3A). Pparγ was even slightly induced in presence of serum only (FCS). The smaller increase of Pparγ after the addition of only IBMX may be due to the absence from the nucleus of the glucocorticoid receptor which must be pre-bound to chromatin for IBMX full activation^25^. Normalization was accomplished by assessing *Vinculin* expression. Similar results were observed also upon partial combinations of the adipogenic inducers.

We have tested the effect of Prep1 DR on other known targets of the adipogenic inducers. The insulin pathway appeared to be activated in P cells as shown by the strong and early phosphorylation of Insulin Receptor Substrate 1 (pIrs1; Fig. 3B) at already 5 min after induction with respect to C cells, that show major phosphorylation after 40 min. Such faster phosphorylation of Irs1 is accompanied by a stronger phosphorylation of Akt kinase at both Thr 308 (Fig. 3C) and Ser 473 (Supplementary Fig. S6A). In addition, the increased expression levels of *Sterol Regulatory Element Binding Transcription Factor 1* (*Srebf1*) in P cells (Fig. 3D) are consistent with activation of the Akt pathway^26,27^.

In the glucocorticoid pathway, dexamethasone-dependent silencing of *Pref1* was faster in P rather than C cells, as shown by its dramatic inactivation at 72 hours (Fig. 3E). Finally, also the cAMP pathway showed earlier and stronger activation. Indeed, even though two very early players of the pathway, *Klf4* and *Egr2* (aka as Krox20) do not change their expression at the RNA level in P as compared to C cells (Fig. 3F and Supplementary Fig. S6B), conversely, *Klf5*, a transcriptional regulator of Pparγ2 and a downstream target of C/EBPβ, is significantly up-regulated in P as compared to C cells, already a few hours after induction (Fig. 3G,H). Figure 3I also shows that the RNA level of *Cebpd* is significantly increased in P cells, as compared to control cells; a similar behavior is shown by the protein (Supplementary Fig. S6C). These results are also confirmed by the RNA-seq data (Supplementary Table S1) that show the increase of the *Cebpd* expression. Overall these data suggest that Prep1 is a general inhibitor of adipogenic differentiation.

In conclusion, it appears that Prep1 DR favors adipogenesis independently of the adipogenic inducer employed, activating essentially many of the adipogenic target genes, and acting at a very early stage preceding the induction of differentiation.

### Prep1 DR strongly increases C/EBPβ binding to chromatin

**Figure 4:**
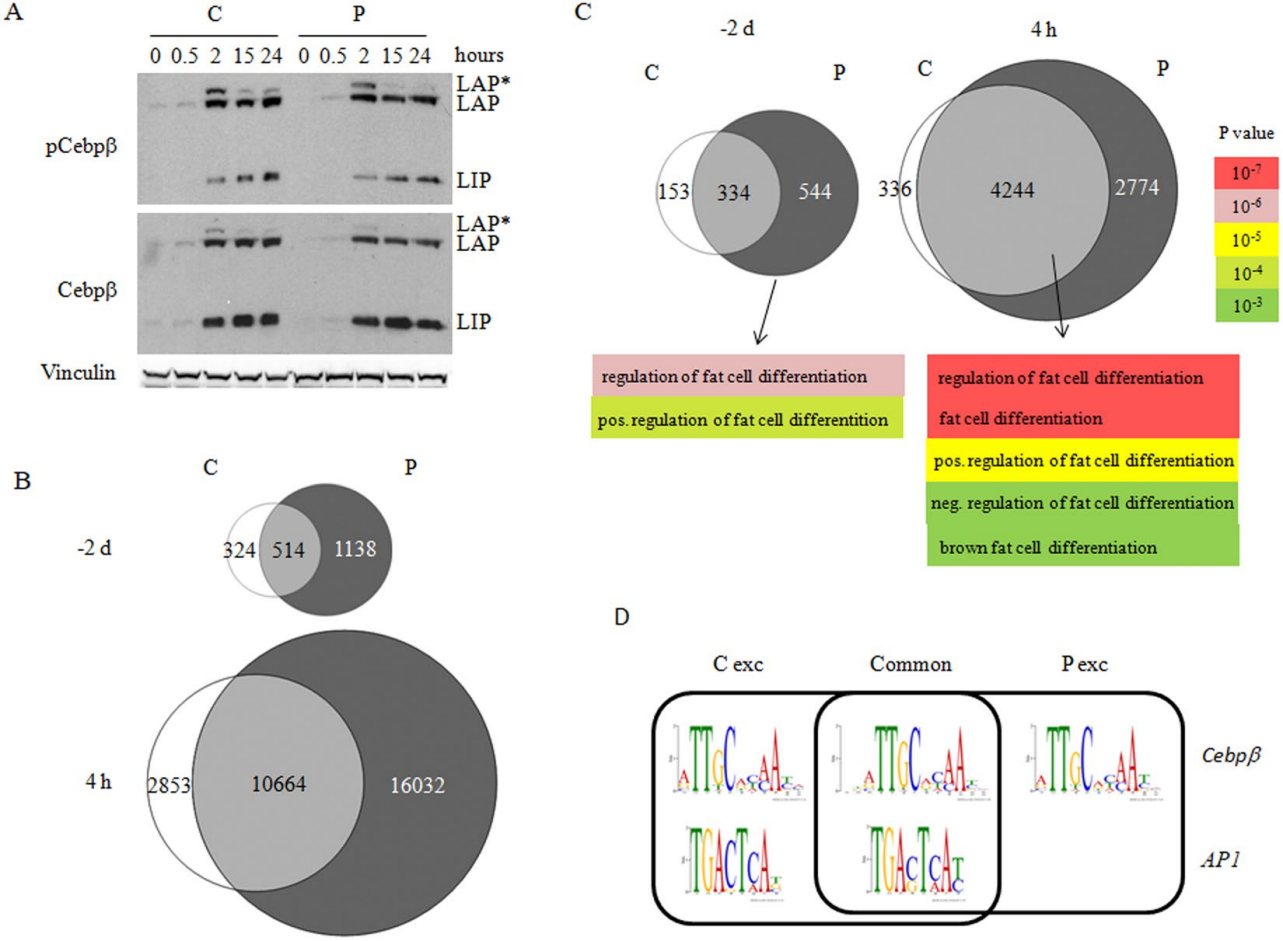
Prep1 down-regulation increases C/EBPβ binding to chromatin in 3T3-L1 cells without affecting C/EBPβ level and phosphorylation. (**A**) Immunoblotting analysis of cell extracts from P and C cells at various time points after induction using anti-phospho-C/EBPβ (Thr235), and total C/EBPβ Abs, recognizing three isoforms of C/EBPβ - LAP*, LAP, LIP. Vinculin Ab was used as loading control. (**B**) Venn diagrams of C/EBPβ binding peaks in Prep1 down-regulated (P) and control (C) cells before (−2 d) and 4 hours (4 h) after the induction of differentiation. (**C**) Venn diagrams and Gene Ontology analysis of genes bound by C/EBPβ in P and C cells before (−2 d) and 4 hours (4 h) after induction. (**D**) DNA-binding motifs (Cebpβ and AP1) of C/EBPβ binding sites in P and C cells before (−2 d) the induction of differentiation, depicting common and exclusive peaks.

C/EBPβ is an essential and very early transcription factor in adipogenesis. Hence, the effects of Prep1 DR might be explained by its increase or activation^28^. The expression of *Cebpb*, however, was not significantly changed upon Prep1 DR as shown by RNA-seq analysis (Supplementary Table S1) and qPCR (Supplementary Fig. S6D). Since C/EBPβ must be phosphorylated in order to acquire its DNA-binding activity^29,30^, we checked whether the levels of total and phosphorylated C/EBPβ isoforms (LAP*, LAP, LIP) were affected by Prep1 DR. However, both total and phosphorylated levels of C/EBPβ (LAP is phosphorylated at Thr188, LIP is phosphorylated at Thr37) were equally affected in P v. C cells, at all times tested after the induction (Fig. 4A).

Since C/EBPβ is the main player in the formation of multifactor complexes recruited on enhancers in chromatin hotspots already a few hours upon induction of differentiation^4^, we analyzed the effect of Prep1 DR on the binding landscape of C/EBPβ in 3T3-L1 cells comparing specific ChIP-seq at day −2 and 4 hours after induction in P v. C cells (accessible in GEO at the accession number GSE100057). The specificity of the C/EBPβ antibody used for ChIP was checked by western blot and proved that C/EBPβ is properly immunoprecipitated (Supplementary Fig. S9). Four hours after induction corresponds to the formation of transcription factor hotspots during adipogenesis^9^. The data obtained through our assays are reported in Table 2. Venn diagrams illustrating the effect of Prep1 DR on C/EBPβ DNA binding are shown in Fig. 4B. Prep1 DR induced a 2-fold increase of highly significant DNA-binding sites (p < 10^−5^) in P versus C cells before differentiation at day −2, and a further 2-fold increase 4 hours after induction of differentiation (Fig. 4B and Supplementary Fig. S10). The increase was distributed among all types of peaks, i.e. transcription start site associated (TSSA), intragenic (IG), close (CI)- and far-intergenic (FI) (Table 2), but their distribution among these categories was not affected. As C/EBPβ is the common transcription factor recruited in the hotspots established at 4 hours after the induction^4^, we tested the correspondence between the C/EBPβ peaks and the hotspots. Table 2 shows that 54% of the C/EBPβ peaks in differentiated control cells are present within hotspots; however, this fraction is increased to 74% in differentiated P cells. We conclude, therefore, that Prep1 DR strongly increases C/EBPβ binding to chromatin; this may occur because the absence of Prep1 provides a genomic environment better suited for C/EBPβ binding to DNA.

**Table 2:** ChIP-seq analysis of the effect of Prep1 DR on Cebpβ binding to DNA in C and P cells*.

	Undifferentiated C cells (−2 days)	Undifferentiated P cells (−2 days)	Differentiated C cells (+4 hrs)	Differentiated P cells (+4 hrs)
Total n. ofpeaks	838	1,652	13,517	26,696
TSSA^#^	58 (7)	91 (6)	484 (4)	989 (4)
IG^#^	309 (37)	652 (39)	5,671 (42)	11,603 (43)
CI^#^	136 (16)	248 (15)	1,991 (15)	3,600 (14)
FI^#^	335 (40)	661 (40)	5,371 (39)	10,504 (39)
number of hotspots overlapping with peaks^$^	NA	NA	6,074 (54)	8294 (74)

*The numbers refer to highly specific peaks (p < 10^−5^). The numbers in brackets show the percent of the peak categories versus total number of peaks.

^#^TSSA: transcription start site associated; IG: intragenic: CI: close intergenic (less than 20 Kb); FI: far intergenic (>20 Kb).

^$^hotspots’ coordinates established by^4^, NA - not available.

Regulatory elements regulate gene expression during adipogenic differentiation of 3T3-L1 cells even when they are Mbs away from a corresponding promoter^6^. We associated each peak located closer than 20 kbs to a transcription start site (TSSA), or located intragenically (IG), to the corresponding gene and found that the number of C/EBPβ-bound genes was also significantly increased in P cells both before and after differentiation (Fig. 4C). Moreover, at time day −2, i.e. before differentiation, GO analysis of the 544 genes bound by C/EBPβ exclusively in P cells (Fig. 4C) was enriched for such GO terms as “Regulation of fat cell differentiation” (p < 10^−6^) and “Positive regulation of fat cell differentiation” (p < 10^−4^). The same GO categories and few others related to adipogenesis (Fat cell differentiation, White fat cell differentiation) were enriched at 4 hours after induction in P cells*;* however, these genes were bound by C/EBPβ also in C cells.

The distribution of the enriched GO categories between P and C cells is shown in Supplementary Table S3, which also shows an overall increase in all peaks’ categories. It is important to notice that virtually none of the genes are exclusively bound by C/EBPβ in control cells before induction. We also checked whether the expression of these genes is changed at day −2 using our RNA-seq data. While more than half of C/EBPβ-bound genes from “Fat cell differentiation” category in P cells was up-regulated, none of the 6 control-specific genes changed their expression (data not shown). This is in agreement with the possibility of Prep1 regulating C/EBPβ access to DNA and hence allowing premature induction of expression of adipogenic genes.

We also analyzed the ChIP-seq C/EBPβ DNA binding motifs using MEME software. While Prep1 DR did not affect C/EBPβ binding to the canonical site^9^, the binding to the additional AP1 consensus sequences was greatly reduced (from 35% to less than 15%) in peaks exclusively bound in P cells (Fig. 4D). This is in line with Prep1 DR regulating C/EBPβ DNA-binding efficiency.

## Discussion

In this paper we have shown that Prep1 DR in 3T3-L1 preadipocytes and in *ex vivo Prep1 *hypomorphic MSCs predisposes to adipogenic differentiation (Figs 1 and 2). First, and most importantly, the effect of Prep1 DR, i.e. the activation of some markers of adipogenic differentiation, precedes the addition of the inducers. Moreover, Prep1 DR did not affect one single pathway activated by insulin, glucocorticoids or cAMP, i.e. the inducers of *in vitro* differentiation. Finally, the major effect of Prep1 DR was an increased binding of C/EBPβ to DNA which, however, was not explained by a direct effect on the level or activation of this protein.

Since Prep1 belongs to a family of proteins which are essential in embryonic development, it was initially considered as a direct regulator of development^16,31^. Although the *Prep1*-deficient phenotype, i.e. the lethality of the Prep1-deficient embryos in different organisms^13–15,18,32^, suggests a developmental function, the effects were never shown to be targeted on developmentally essential genes. Absence of Prep1 causes phenotypes that may be attributed to basic cellular activities, such as apoptosis and DNA damage^18^; however, differentiation of several cell lineages was observed^13,14,33^, implying that differentiation mechanisms may be altered. Nevertheless, the analysis of the DNA binding landscape displayed by Prep1 did not detect any distinct involvement in development/differentiation-related genes. Rather, the Gene Ontology analysis of the genes bound and regulated by Prep1 in E11.5 mouse embryos identified gene categories that belong to “basic cellular machineries or function” such as transcription regulation, DNA metabolism, signaling and chromatin modifications^12^. In addition, while *in vivo* Prep1 DR is not necessarily incompatible with life (*Prep1* ^*i/i *^hypomorphic mice show a partially-penetrant phenotype)^14,23^, induction of differentiation of Prep1 DR ES cells has shown dramatic effects^19^.

Therefore, we proposed that also the phenotype of Prep1 DR 3T3-L1 and of *Prep1* ^*i*/*i *^mesenchymal adipogenic progenitors may be considered under this angle. The phenotypes observed in both systems appear to be secondary to a fundamental effect, which is already in place before the induction of differentiation, and becomes evident only after the addition of the differentiation cocktail. We hypothesize that Prep1 DR modifies chromatin organization. For example, the increased binding landscape of C/EBPβ might be due to the presence of a more accessible, Prep1-dependent, chromatin architecture. As Prep1 is a regular transcriptional partner of Pbx1 and since Pbx1 DR also shows a similar phenotype to Prep1 DR, one wonders how much of the Pbx1 function is due to a specific differentiation function and how much is due to more basic cellular activities.

In conclusion, we have shown for the first time that Prep1 transcription factor is required for adipogenic differentiation of both a preadipocytic cell line and of *ex vivo* mesenchymal progenitor cells. It performs its action indirectly, inducing an extensive DNA binding of C/EBPβ at the early stages of the adipogenic process. The mechanism of this induction remains to be elucidated.

## Methods

### *In vitro* cultures of Mesenchymal Stromal Cells (MSCs) and 3T3-L1 cells

Mice were sacrificed by cervical dislocation before collecting tibias and femurs. Bone marrow cells were flushed and seeded in culture using MesenCult Basal Medium supplemented with 20% Mesenchymal Mouse Stimulatory Supplement (StemCell Technologies) and 1% Pen-Strept (Life Technologies) (Complete Medium). Cells were grown at 37 °C in humidified atmosphere at 5% CO_2_. Medium was changed every 3 days and cells were trypsinized at confluence and reseeded at 2 × 10^4^ cells/cm^2^ (passage 1, p1). All experiments were performed at passage 2 (p2). All the experimental protocols on mice were conducted in compliance with DL 26/2014: implementation of European Directive 2010/63 on the protection of animals used for scientific purposes. All animal experiments were approved by the Institutional Animal Care and Use Committee of the CNR Research Campus in Pisa.

3T3-L1 cells were cultured in DMEM medium containing 10% BCS. Prep1 down-regulated 3T3-L1 cells were obtained by infection with a pLKO.1-based lentivirus containing Prep1-specific shRNA1 (5′ - AACCAGTTGTTGACTTGAAGC - 3′) or shRNA2 (5′-TTGCTTCCTGACAAAGTTCTC-3′) and control cells were infected with a scrambled shRNA. Cells were selected in puromycin for 3 days. The level of Prep1 expression was verified by immunoblotting using anti-Prep1 monoclonal B2 antibody (sc-25282, Santa Cruz Biotechnology, Santa Cruz, USA).

### Adipogenic differentiation

MCSs were seeded at p2 at 2 × 10^4^ cells/cm2 and at 80% confluence the Complete Medium was replaced with Adipogenic Induction Medium (MesenCult Basal Medium supplemented with 20% AdipogenicStimulatory Supplement (StemCell Technologies) and 1% Pen-Strept (Life Technologies) we followed the manufacturer’s recommendations.

For adipogenic differentiation of 3T3-L1 cells, two days after confluence the medium was changed to differentiation medium I containing 1 μM dexamethasone, 0.5 mM 3-isobutyl-1-methylxanthine, and 1 μg/ml insulin in DMEM containing 10% of FBS. Two days after the induction of differentiation the medium was changed do differentiation medium II containing only 1 μg/ml insulin in the presence of 10% of FBS. After 4 days of differentiation the medium was changed to differentiation medium III containing only 10% of FBS.

### Oil Red O staining

Differentiated 3T3-L1 and MSC cells were stained by Oil-red O using a standard protocol^34^. Briefly, cells were fixed in 3.7% of formaldehyde in PBS for 30 min at room temperature and then stained in 0.3% of Oil Red O in 60% of isopropanol for 30 min at room temperature. For quantification assays, Oil Red O was extracted from the stained cells by using 100% isopropanol and absorption was measured at 490 nm.

### ChIP-seq

Chromatin immunoprecipitations (IP) were performed using standard methods with anti-C/EBPβ C19 antibody (sc-150, Santa Cruz Biotechnology, Santa Cruz, USA). Cells were cross-linked in complete medium (10% FBS) containing 1% formaldehyde for 10 min, and glycine was added to stop the reaction (125 mM final concentration). Fixed cells were washed three times (5 min each) in cold PBS and lysed in LB1 buffer (containing 0.5% NP-40 and 0.25% triton X-100). Nuclei were washed in LB2 buffer (10 mM Tris-HCl pH 8 and 200 mM NaCl) to remove detergents and resuspended in LB3 buffer (containing 0.1% Na-deoxycholate and 0.5% N-lauroylsarcosine). Chromatin was sonicated in Covaris ultrasonicator to obtain fragments ranging 150–250 bps. Sonicated chromatin was incubated with antibody-bound protein G-conjugated magnetic beads (Invitrogen, Carlsbad, USA). For each IP we used 20 μg of  antibody. IP with rabbit IgG was performed as negative control. After overnight IP at 4 °C the bound complexes were washed twice in WB1 (50 mM Hepes-KOH pH 7.5, 140 mM NaCl, 1 mM EDTA, 1% Triton-X100, 0.1% Na-doexycholate), twice in WB2 (50 mM Hepes-KOH pH 7.5, 500 mM NaCl, 1 mM EDTA, 1% Triton-X100, 0.1% Na-deoxycholate) and twice in LiCl WB (10 mMTris-Cl pH 8.0, 250 mMLiCl, 0.5% NP-40, 0.5% Na-deoxycholate, 1 mM EDTA). Immunoprecipitated complexes were eluted from the beads by incubation for 30 min in EB (2% SDS in TE) at 65 °C. The eluted material was reverse cross-linked at 65 °C overnight and incubated for 1 h at 55 °C with proteinase K. DNA was purified with a PCR purification kit (Qiagen, Netherlands). ~10 ng of immunoprecipitated DNA was processed for sequencing. After library preparation DNA was sequenced using an Ion-Torrent system. After eliminating artifacts with FASTX-Toolkit v.0.0.13.2, reads were aligned to the mm9 genome using Bowtie v.0.12.8 (3), allowing up to two mismatches per read and discarding reads with more than one mapping. Duplicate reads were removed using Samtoolrmdup v 0.1.18. Then we down-sampled mapped reads to 8 mln using Samtools v 1.3.1.

To identify enriched domains we used MACS version 2.0.10.20131028 with default parameters^35^, except for p-value threshold (10e-5).The reads were mapped against the mm9 mouse genome. De novo motif discovery was run to identify consensus sequences enriched in the selected regions versus the whole genome using MEME-SUITE as de novo motif finder algorithm^36^.

### RNA-seq

For RNA-seq, total RNA was purified and the library prepared. For each time point (−2 days and 1 day) and sample (control and Prep1 down-regulated) we used 2 biological replicas. The sequencing was performed using Ion-Torrent system. After eliminating artifacts with FASTX-Toolkit v.0.0.13.2, reads were aligned to the mm9 reference using Tophat v2.0.9. Then GenomicFeatures, GenomicRanges and GenomicAligments R libraries were used to count the number of reads with respect to the annotation reference (UCSC, genome = ‘mm9’, tablename = ‘refGene’). Differentially expressed genes between different conditions were retrieved using DESEQ R library^37^, filtering the results using padj <0.1 as a threshold. Gene ontology analysis was performed using Gorilla software^38^.

The heat maps were drawn plotting the ratios between each of normalized RPKM versus the average of two control replicas at each time point and sample.

### qPCR and immunoblotting

RNA extraction was processed according to the RNeasy (QIAGEN) protocol. After genomic DNA degradation with the RNase-Free DNase kit (QIAGEN), reverse transcription was performed with the Superscript III (Invitrogen). For *Srebf1*, *Klf4*, *Klf5*, *Krox20*, *C*/*EBP*δ, *C*/*EBP*β, *Prep1*, *Glut4* expression analysis, cDNAs were subjected to qRT-PCR on a Roche LightCycler480 (Roche) using predesigned primers (RealTime ready assays, Roche). Sequences are available upon request. Results were normalized to *Gapdh* gene expression.

Antibodies used were anti-Prep1 (Santa Cruz sc-25282), anti-C/EBPα (D56F10 Cell signaling Technology #8178), anti-C/EBPβ (sc-150, Santa Cruz Biotechnology, Santa Cruz, USA), anti-phospho-C/EBPβ (Thr235) (Cell Signaling Technology #3084), anti-C/EBPδ (sc-151, Santa Cruz Biotechnology, Santa Cruz, USA), anti-Pparγ (81B8, Cell signaling Technology #2443), anti-pIrs1 (Tyr941) (07-848-I Millipore), anti-pAkt (Ser473) (D9E Cell signaling Technology #4060), anti-pAkt (Thr308) (D25E6 Cell signaling Technology #13038), anti-Klf5 (Abcam ab24331), anti-Vinculin (Sigma V9131).

### Data availability

The datasets generated during the current study are available in the GEO (accession number GSE100057).

## Electronic supplementary material

Supplementary information

## References

[1] Spalding KL (2008). Dynamics of fat cell turnover in humans. Nature.

[2] Jeffery E, Church CD, Holtrup B, Colman L, Rodeheffer MS (2015). Rapid depot-specific activation of adipocyte precursor cells at the onset of obesity. Nat Cell Biol.

[3] Berry R, Jeffery E, Rodeheffer MS (2014). Weighing in on adipocyte precursors. Cell Metab.

[4] Siersbaek R (2011). Extensive chromatin remodelling and establishment of transcription factor ‘hotspots’ during early adipogenesis. EMBO J.

[5] Siersbaek R, Nielsen R, Mandrup S (2012). Transcriptional networks and chromatin remodeling controlling adipogenesis. Trends Endocrinol Metab.

[6] Siersbaek M (2017). High fat diet-induced changes of mouse hepatic transcription and enhancer activity can be reversed by subsequent weight loss. Sci Rep.

[7] Fu M (2005). A Nuclear Receptor Atlas: 3T3-L1 adipogenesis. Mol Endocrinol.

[8] MacDougald OA, Lane MD (1995). Transcriptional regulation of gene expression during adipocyte differentiation. Annu Rev Biochem.

[9] Siersbaek R (2014). Molecular architecture of transcription factor hotspots in early adipogenesis. Cell Rep.

[10] Lefterova MI, Haakonsson AK, Lazar MA, Mandrup S (2014). PPARgamma and the global map of adipogenesis and beyond. Trends Endocrinol Metab.

[11] Monteiro MC (2011). PBX1: a novel stage-specific regulator of adipocyte development. Stem Cells.

[12] Penkov D (2013). Analysis of the DNA-binding profile and function of TALE homeoproteins reveals their specialization and specific interactions with Hox genes/proteins. Cell Rep.

[13] Fernandez-Diaz LC (2010). The absence of Prep1 causes p53-dependent apoptosis of mouse pluripotent epiblast cells. Development.

[14] Ferretti E (2006). Hypomorphic mutation of the TALE gene Prep1 (pKnox1) causes a major reduction of Pbx and Meis proteins and a pleiotropic embryonic phenotype. Mol Cell Biol.

[15] Di Rosa P (2007). The homeodomain transcription factor Prep1 (pKnox1) is required for hematopoietic stem and progenitor cell activity. Dev Biol.

[16] Berthelsen J, Zappavigna V, Mavilio F, Blasi F (1998). Prep1, a novel functional partner of Pbx proteins. EMBO J.

[17] Berthelsen J, Kilstrup-Nielsen C, Blasi F, Mavilio F, Zappavigna V (1999). The subcellular localization of PBX1 and EXD proteins depends on nuclear import and export signals and is modulated by association with PREP1 and HTH. Genes Dev.

[18] Deflorian G (2004). Prep1.1 has essential genetic functions in hindbrain development and cranial neural crest cell differentiation. Development.

[19] Laurent A (2015). ChIP-Seq and RNA-Seq analyses identify components of the Wnt and Fgf signaling pathways as Prep1 target genes in mouse embryonic stem cells. PLoS One.

[20] Dardaei L (2015). Tumorigenesis by Meis1 overexpression is accompanied by a change of DNA target-sequence specificity which allows binding to the AP-1 element. Oncotarget.

[21] Blasi, F., Bruckmann, C., Penkov, D. & Dardaei, L. A tale of TALE, PREP1, PBX1, and MEIS1: Interconnections and competition in cancer. *Bioessays*, doi:10.1002/bies.201600245 (2017). 10.1002/bies.201600245 28322463

[22] Penkov D (2005). Involvement of Prep1 in the alphabeta T-cell receptor T-lymphocytic potential of hematopoietic precursors. Mol Cell Biol.

[23] Longobardi E (2010). Prep1 (pKnox1)-deficiency leads to spontaneous tumor development in mice and accelerates EmuMyc lymphomagenesis: a tumor suppressor role for Prep1. Mol Oncol.

[24] Helledie T (2002). The gene encoding the Acyl-CoA-binding protein is activated by peroxisome proliferator-activated receptor gamma through an intronic response element functionally conserved between humans and rodents. J Biol Chem.

[25] Pantoja C, Huff JT, Yamamoto KR (2008). Glucocorticoid signaling defines a novel commitment state during adipogenesis *in vitro*. Mol Biol Cell.

[26] Lee YS (2007). Chromatin remodeling complex interacts with ADD1/SREBP1c to mediate insulin-dependent regulation of gene expression. Mol Cell Biol.

[27] Ayala-Sumuano JT (2011). Srebf1a is a key regulator of transcriptional control for adipogenesis. Sci Rep.

[28] Steger DJ (2010). Propagation of adipogenic signals through an epigenomic transition state. Genes Dev.

[29] Piwien-Pilipuk G, MacDougald O, Schwartz J (2002). Dual regulation of phosphorylation and dephosphorylation of C/EBPbeta modulate its transcriptional activation and DNA binding in response to growth hormone. J Biol Chem.

[30] Cristancho AG, Lazar MA (2011). Forming functional fat: a growing understanding of adipocyte differentiation. Nat Rev Mol Cell Biol.

[31] Moens CB, Selleri L (2006). Hox cofactors in vertebrate development. Dev Biol.

[32] Modica L, Iotti G, D’Avola A, Blasi F (2014). Prep1 (pKnox1) regulates mouse embryonic HSC cycling and self-renewal affecting the Stat1-Sca1 IFN-dependent pathway. PLoS One.

[33] Micali N, Ferrai C, Fernandez-Diaz LC, Blasi F, Crippa MP (2009). Prep1 directly regulates the intrinsic apoptotic pathway by controlling Bcl-XL levels. Mol Cell Biol.

[34] Kraus NA (2016). Quantitativeassessment of adipocyte differentiation in cell culture. Adipocyte.

[35] Zhang Y (2008). Model-based analysis of ChIP-Seq (MACS). Genome Biol.

[36] Bailey TL (2009). MEME SUITE: tools for motif discovery and searching. Nucleic Acids Res.

[37] Anders S, Huber W (2010). Differential expression analysis for sequence count data. Genome Biol.

[38] Eden E, Navon R, Steinfeld I, Lipson D, Yakhini Z (2009). GOrilla: a tool for discovery and visualization of enriched GO terms in ranked gene lists. BMC Bioinformatics.

