# Supplementary material for: Prep1 prevents premature adipogenesis of mesenchymal progenitors
Source: Sci Rep. 2017 Nov 14;7:15573. doi: 10.1038/s41598-017-15828-1 (PMC5686065; doi:10.1038/s41598-017-15828-1)
Supplement: Supplementary file 1 — Supplementary information [file 41598_2017_15828_MOESM1_ESM.pdf]

Prep1 prevents premature adipogenesis of mesenchymal progenitors

Maroni[1#§]G, Tkachuk[2]V, Egorov[2] A, Morelli[3] MJ, Luongo[1°] R, Levantini[1,4]E, Blasi[5]F, Magli[1] MC and Penkov[5,2,6]* D.

1.Institute of Biomedical Technologies, National Research Council (CNR), Via Moruzzi, 1, 56124 Pisa, Italy.

2. Lomonosov Moscow State University, Leninskiye Gory 1, 119991, Moscow, Russia.

3. Center for Genomic Science of IIT@SEMM, Fondazione Istituto Italiano di Tecnologia (IIT), via Adamello 16, 20139 Milano, Italy

4. Beth Israel Deaconess Medical Center, 330 Blackfan Circle, CLS 428, Boston 02215 MA, USA

5. IFOM (Fondazione FIRC Institute of Molecular Oncology), via Adamello 16, 20139, Milano Italy.

6. National Medical Research Center for Cardiology, 3rd Cherepkovskaya Street 15A, 121552, Moscow, Russia.

§ Present address: Beth Israel Deaconess Medical Center, 330 Blackfan Circle, CLS 428, Boston 02215 MA, USA

° Present address: European Institute of Oncology, via Adamello 16, 20139, Milano, Italy.

*corresponding author: Dmitry Penkov

dmitry.penkov@ifom.eu

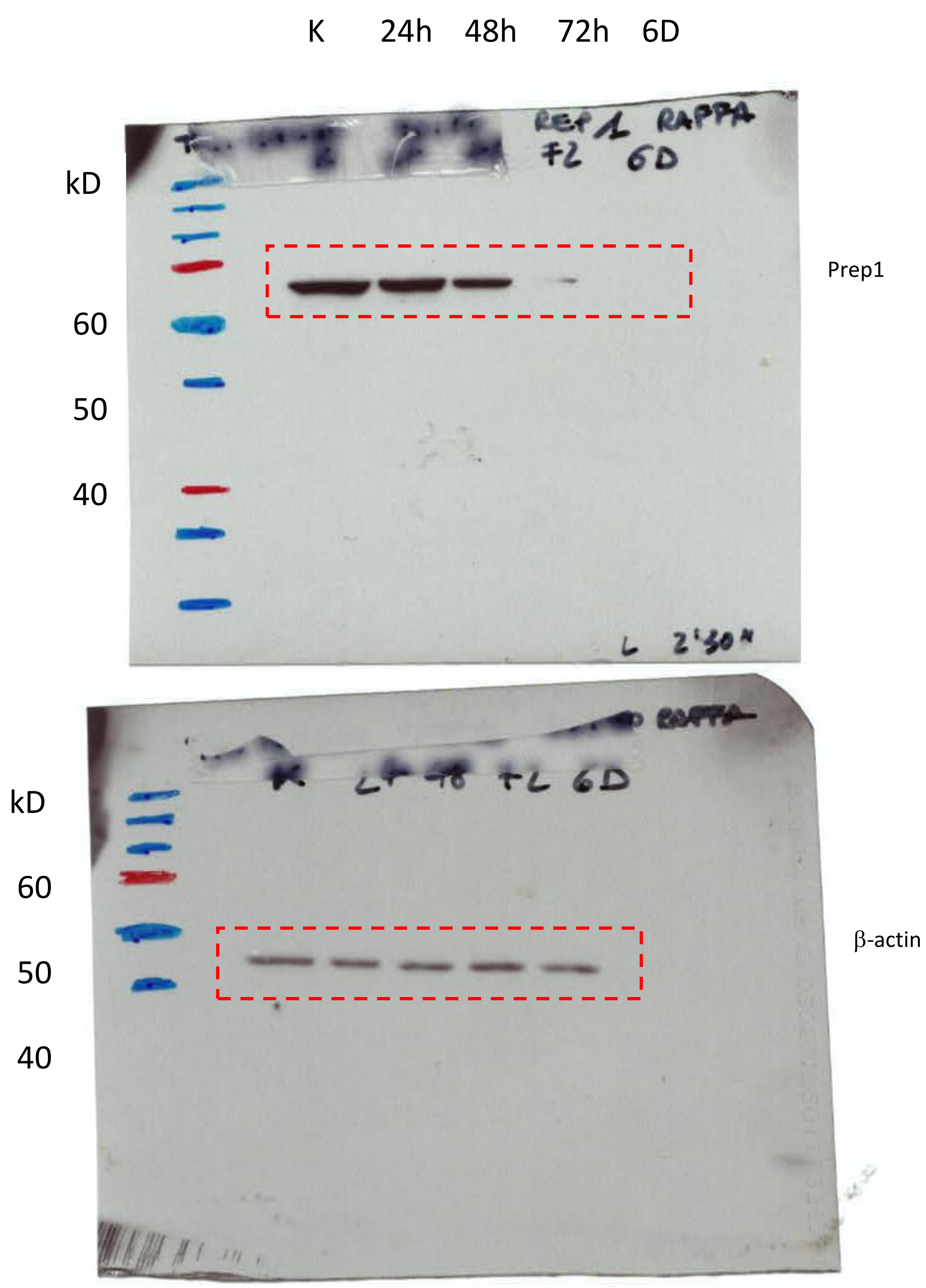

Figure S1. Full-length blots for Figure 1A.

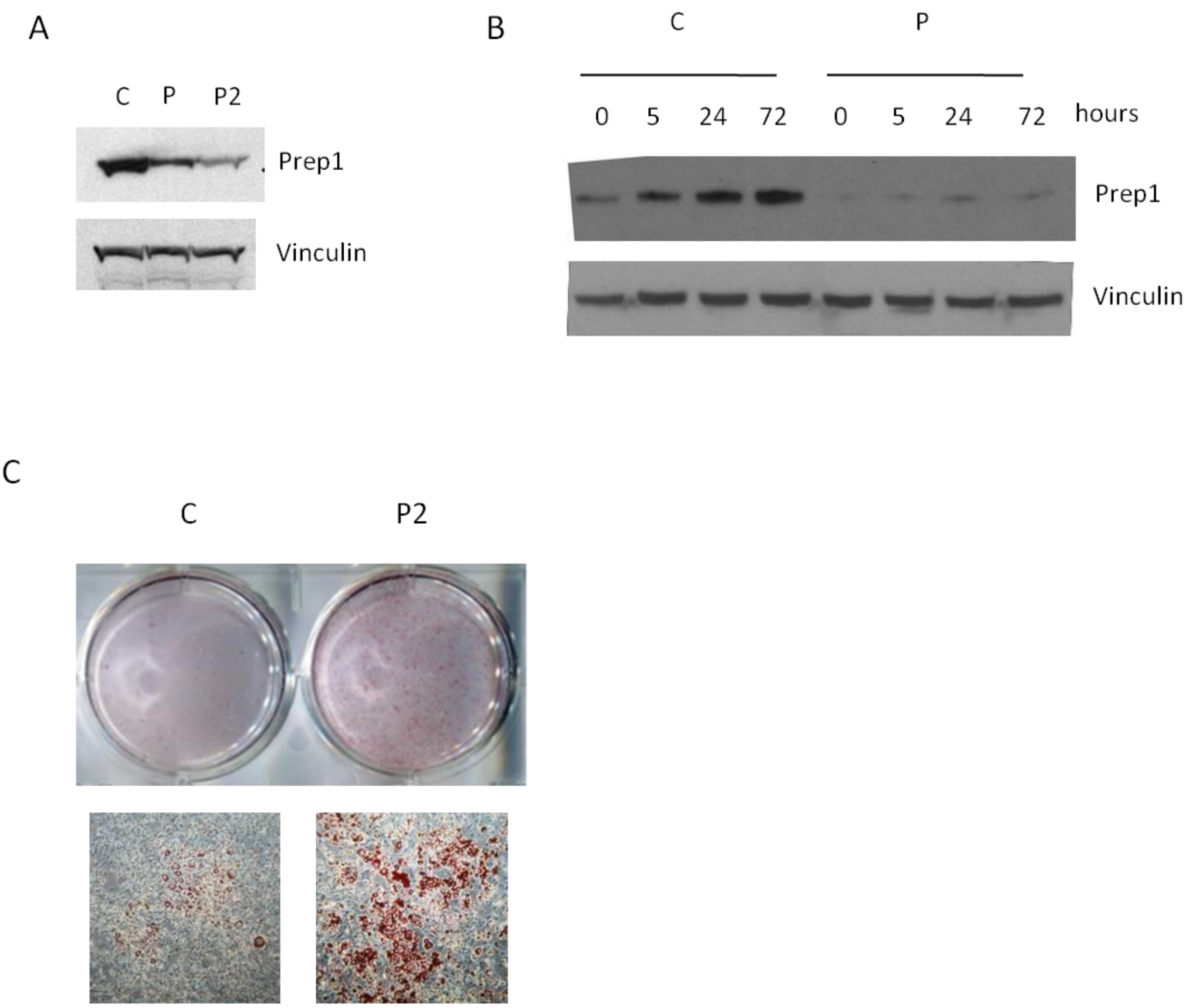

Figure S2. Prep1 down-regulationwith an additional Prep1 shRNA (P2) increases adipogenic differentiationin 3T3-L1 cells.

A. Immunoblotting analysis of cell extracts from C, P and P2 cells before differentiation using anti-Prep1 Ab. Vinculin Ab was used as loading control. B. Immunoblotting analysis of cell extracts from C and P cells at different time points after induction using anti-Prep1 Ab. Vinculin Ab was used as loading control.C. Oil-red O staining of 3T3-L1 control (C) and Prep1 down-regulated (P2) cells using shRNA2 6 days after induction. In the bottom panels the cells are shown at a greater magnification.

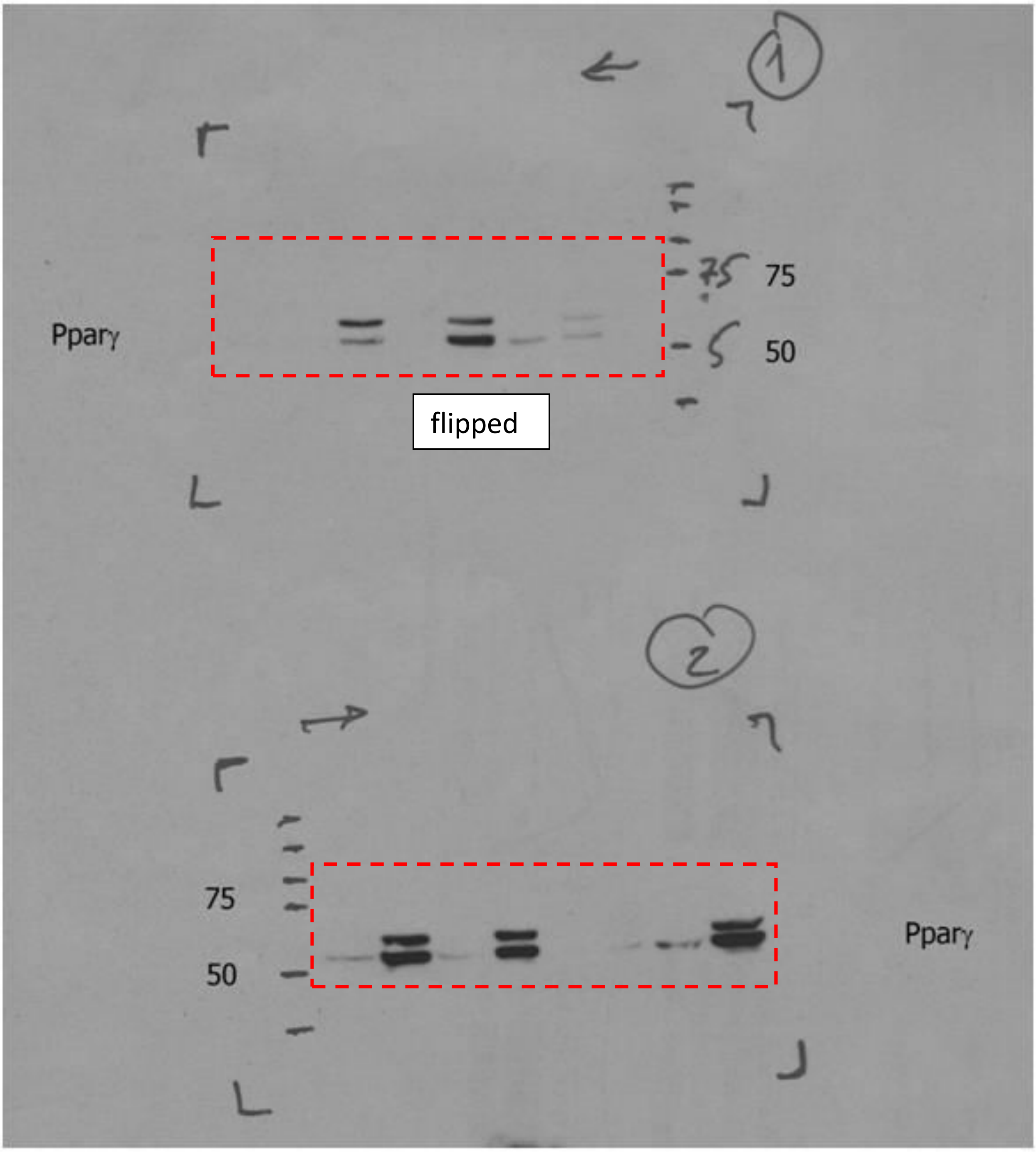

Figure S3. Full-length blots for Figure 3A.

phospho Irs1 (Tyr941

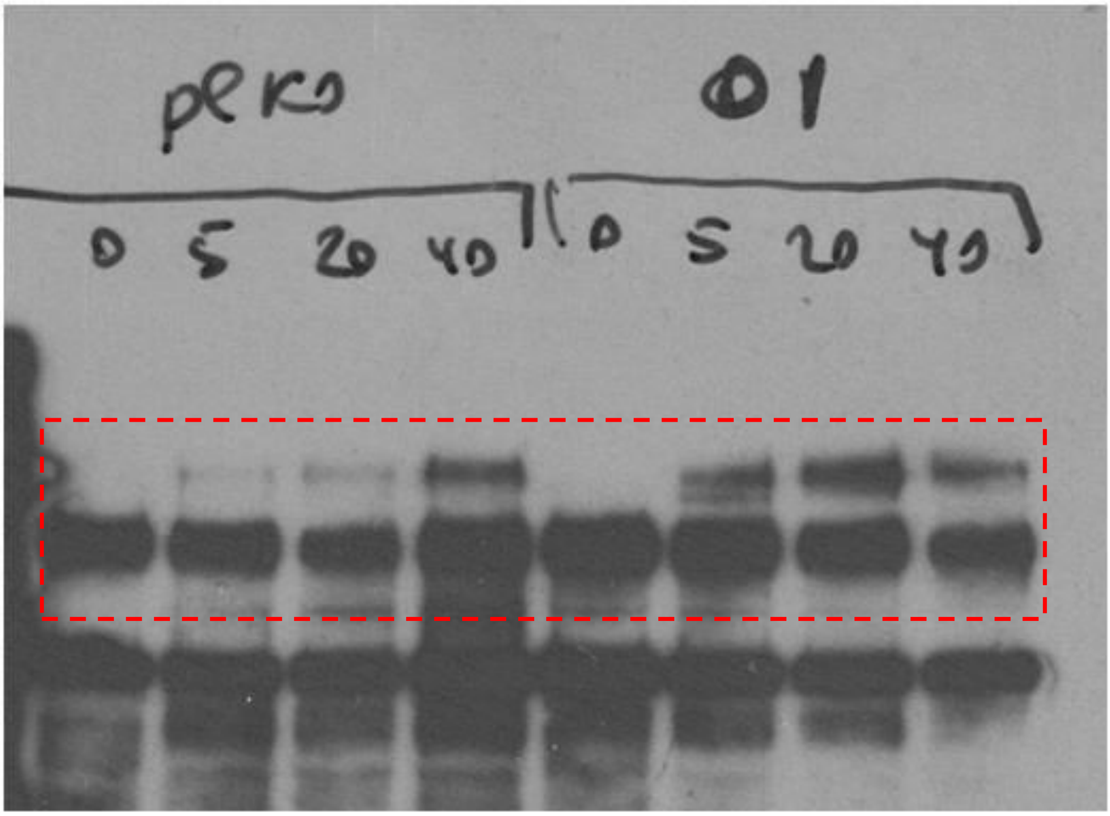

vinculin

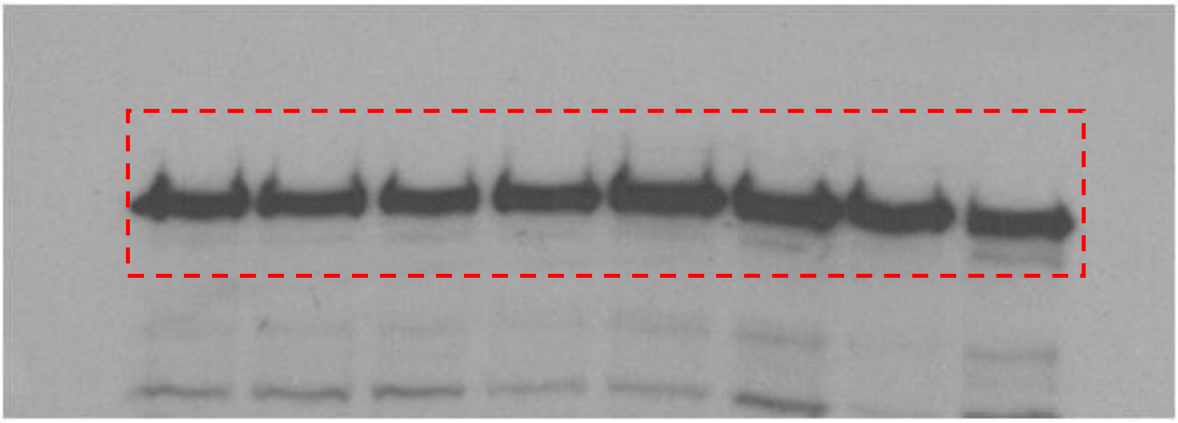

Figure S4. Full-length blots for Figure 3B.

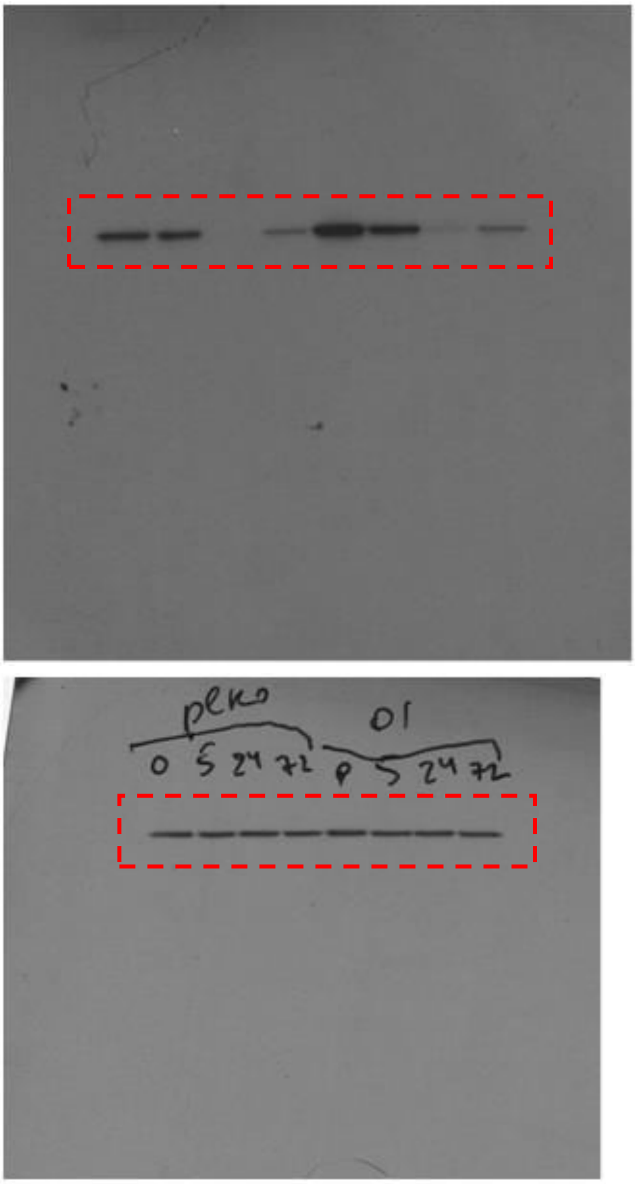

pAkt(thr308)

total Akt

Figure S5. Full-length blots for Figure 3C.

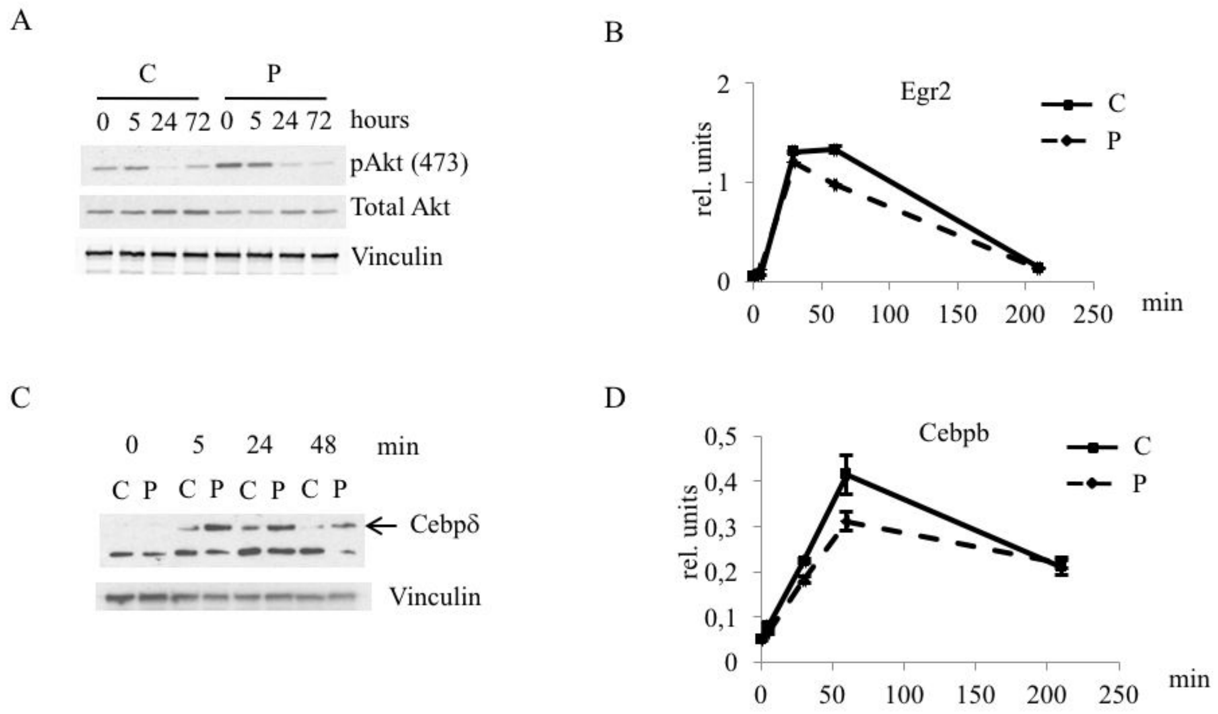

Figure S6. Expression analysis of genes involved in adipogenic differentiation

A. Immunoblotting analysis of cell extracts from P and C cells at various time points after induction using anti phosphoAkt (Thr308) Ab. Total Akt and Vinculin Abs were used as loading control. **B.** qRT-PCR analysis of *Egr2* expression in P (solid line) and C (dashed line) cells at different time points after induction. Relative gene expression was normalized to *Gapdh* expression. **C.** Immunoblotting of cell extracts from P and C cells at various time points after induction using anti-C/EBPδ Ab. Vinculin Ab was used as loading control. **D.** qRT-PCR analysis of *Cebpb* expression in P (solid line) and C (dashed line) cells at different time points after induction. Gene expression was normalized to *Gapdh* levels.

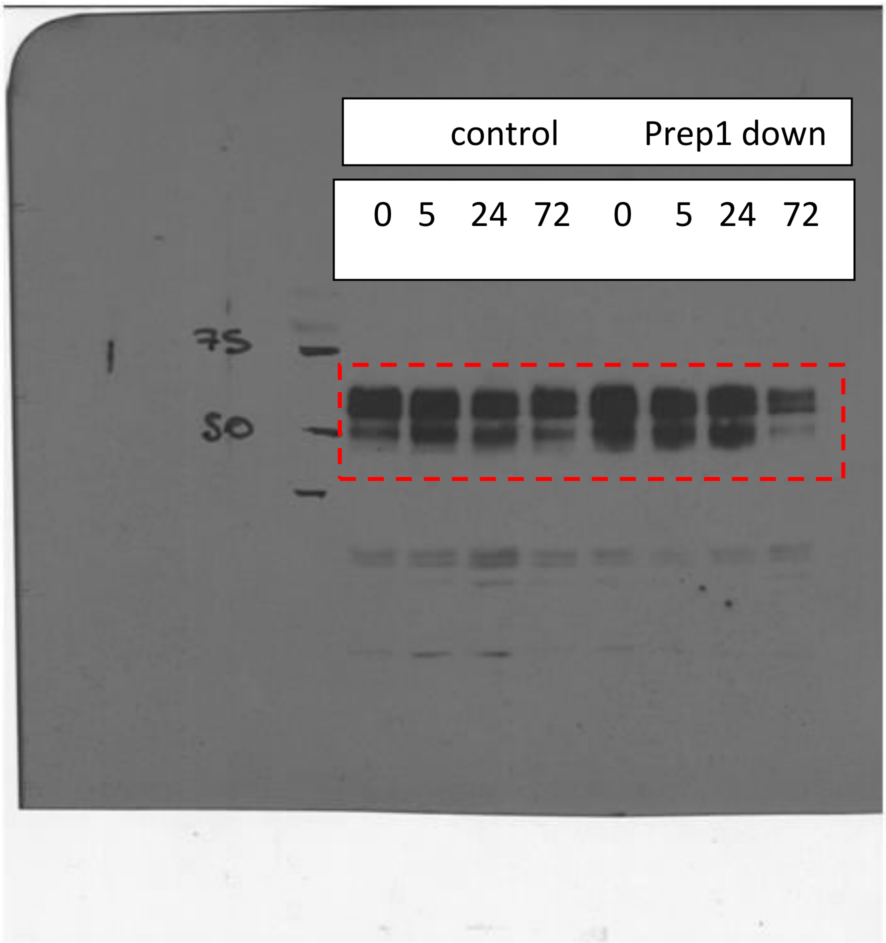

Pref1

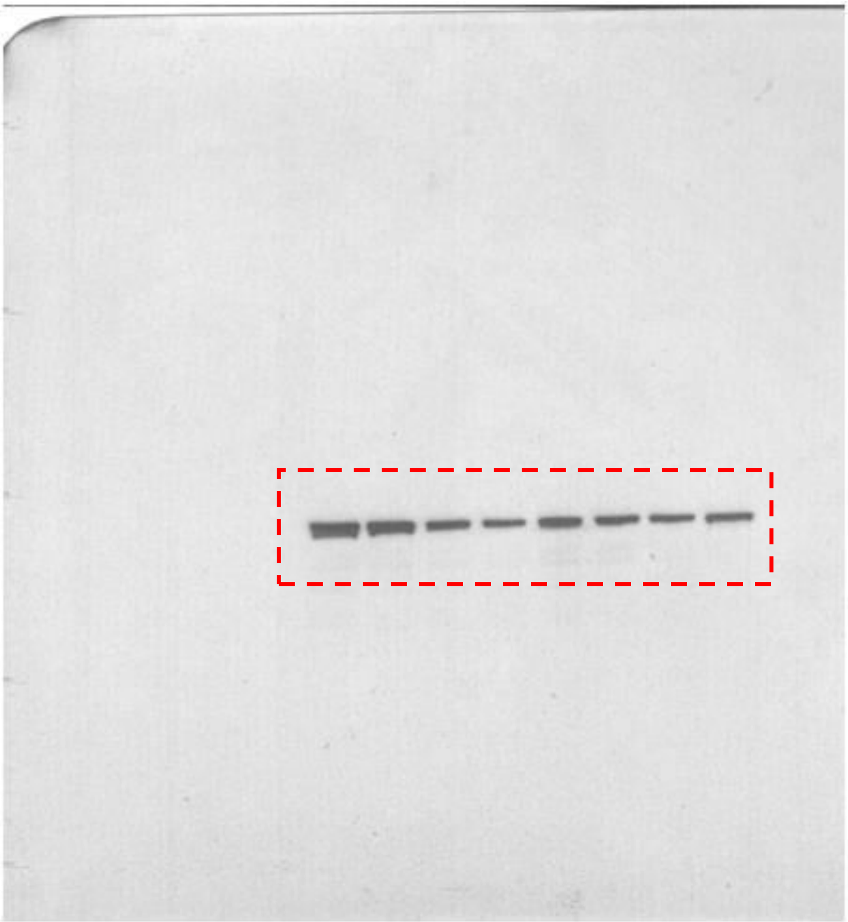

Vinculin

Figure S7. Full-length blots for Figure 3E.

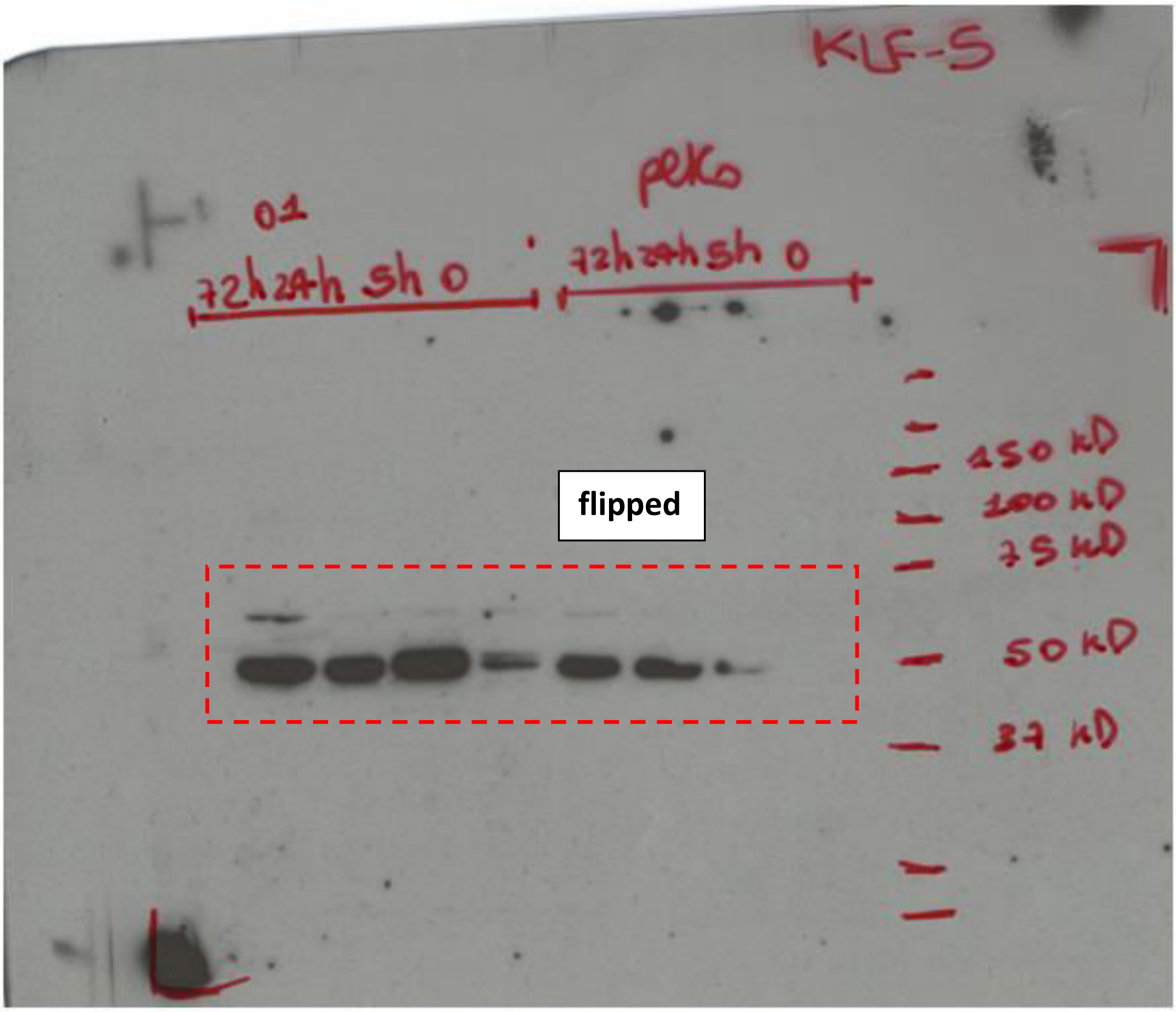

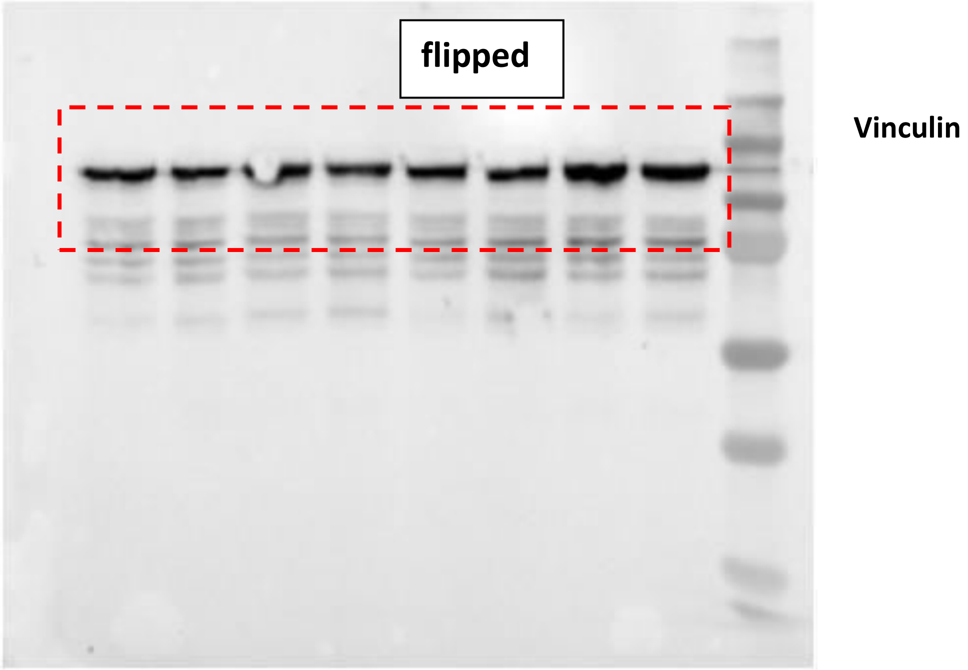

Figure S8. Full-length blots for Figure 3H.

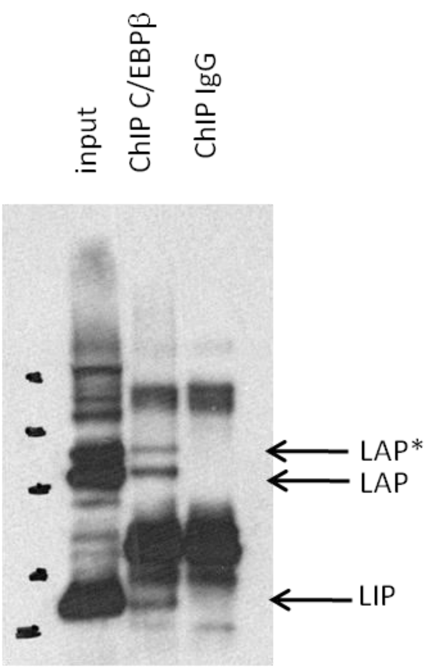

Figure S9. Western blotting of the immunoprecipitated material (ChIP) using anti- C/EBPβ Ab.

Immunoblotting analysis of de-crosslinked input that was used for ChIP in our experiments (input), immunoprecipitated material after ChIP using C/EBPβ (C19) Ab (ChIP C/EBPβ) and rabbit IgG (ChIP IgG). LAP*, LAP and LIP are different isoforms of C/EBPβ protein.

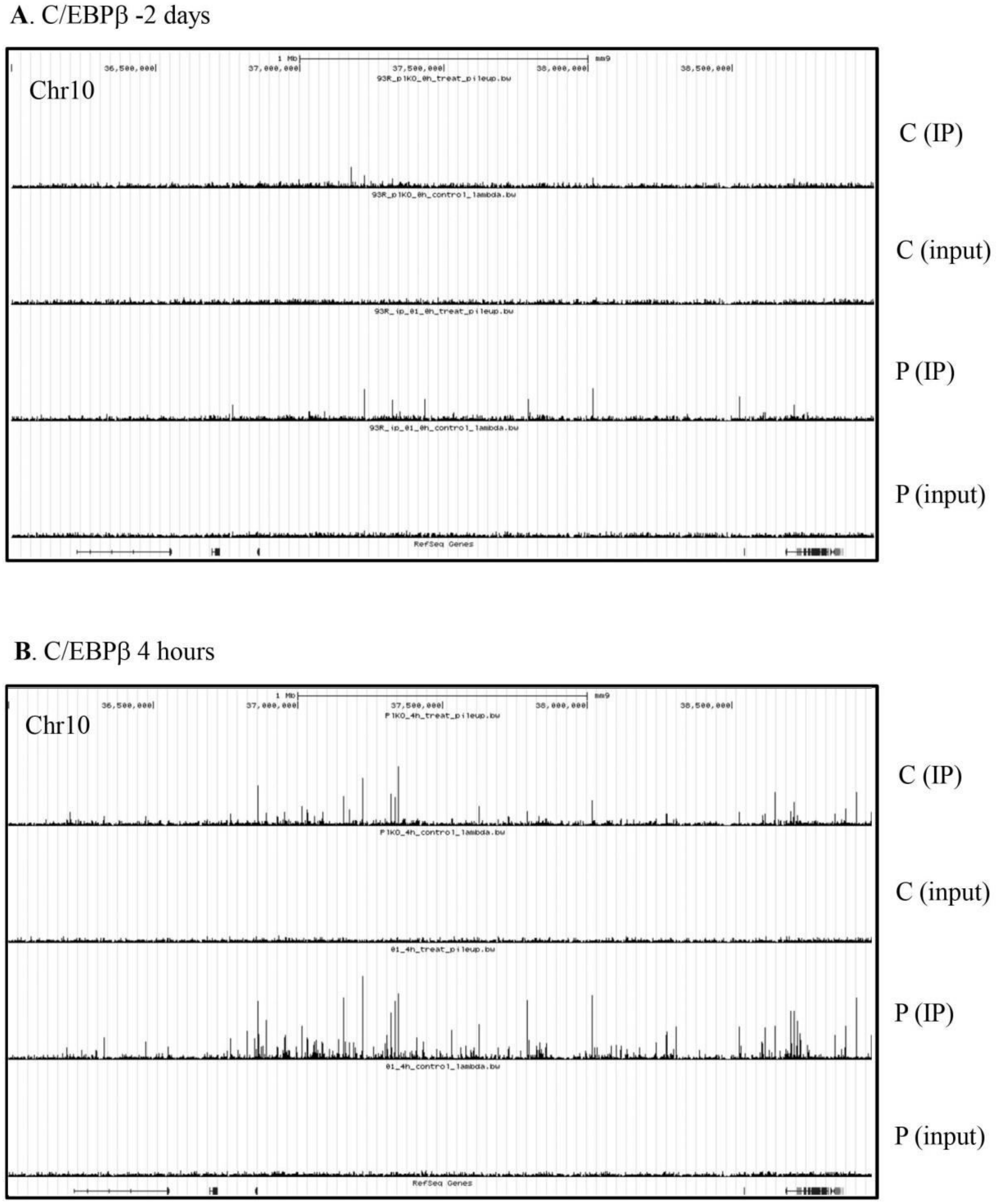

Figure S10.

Examples of C/EBPβ binding peaks in P and C cells before (-2 days) (A) and 4 hours after (B) differentiation induction. IP - immunoprecipitated material, input - non-immunoprecipitated control.

**Gene symbol**	**Fold change**[#]	**P value**	**P adj**[$]
Igfbp5	466.50	6.13E-44	1.33E-39
Islr	33.87	7.48E-23	4.06E-19
Sorcs1	21.56	3.15E-28	2.28E-24
Tusc5	19.01	7.60E-08	1.13E-05
Hp	16.95	3.98E-14	2.78E-11
Col15a1	15.46	1.90E-17	3.43E-14
Lims2	15.43	3.92E-11	1.27E-08
Il31ra	14.99	2.16E-12	1.09E-09
Fibin	14.67	2.55E-10	6.82E-08
Usp18	14.64	4.50E-22	1.95E-18
Nrxn1	14.19	4.42E-10	1.12E-07
Psmb8	14.11	5.42E-17	7.84E-14
Oas2	13.87	8.82E-15	7.66E-12
Ppfia2	12.62	9.98E-15	8.02E-12
Actg2	12.61	3.67E-15	3.32E-12
Gbp6	12.60	7.03E-13	4.01E-10
Psmb9	12.32	2.15E-10	5.82E-08
Rgcc	11.13	3.11E-11	1.02E-08
Ifi27l2a	10.96	1.32E-08	2.33E-06
Cyp2f2	10.95	4.98E-04	2.23E-02
Aldh1a7	10.84	8.50E-08	1.25E-05
Col11a1	10.59	1.68E-04	9.39E-03
Gas6	10.14	2.78E-20	8.61E-17
Tagln	10.11	6.09E-13	3.77E-10
Irf7	10.07	1.55E-15	1.53E-12
Tnn	9.98	1.83E-16	2.20E-13
Mx2	9.86	1.24E-14	9.59E-12

Gbp3	9.14	2.07E-14	1.55E-11
Isg15	9.05	3.68E-17	5.70E-14
Notch3	8.98	2.78E-06	2.85E-04
Dhrs3	8.83	4.30E-09	8.48E-07
Fam71e1	8.72	8.63E-04	3.47E-02
Ggt7	8.70	8.65E-13	4.81E-10
Adamts9	8.38	3.16E-17	5.28E-14
Myl9	8.34	9.49E-04	3.73E-02
Phf11d	8.33	1.43E-05	1.17E-03
Col23a1	7.90	7.96E-12	3.26E-09
Fst	7.75	3.29E-16	3.75E-13
Apod	7.66	2.16E-08	3.61E-06
Nr4a3	7.64	5.76E-05	3.88E-03
Gbp2	7.55	1.02E-13	6.92E-11
Kiss1r	7.47	5.87E-04	2.56E-02
Gm4951	7.27	1.71E-06	1.94E-04
Tap2	7.25	3.25E-04	1.61E-02
Igtp	7.16	1.10E-11	4.26E-09
Oas1a	6.99	2.77E-11	9.39E-09
Prkg1	6.88	2.86E-06	2.91E-04
Ifi44	6.84	7.53E-12	3.14E-09
Bcl11b	6.64	2.48E-03	8.10E-02
Cebpa	6.60	1.15E-09	2.65E-07
Oas1g	6.54	1.39E-05	1.14E-03
Iigp1	6.50	8.38E-10	2.00E-07
Cebpd	6.26	4.08E-12	1.92E-09
Fos	6.26	1.94E-11	7.00E-09
Btg2	5.93	2.23E-09	4.84E-07
Brinp3	5.85	3.40E-10	8.78E-08

Tap1	5.65	9.80E-12	3.87E-09
Acta2	5.60	4.35E-08	6.74E-06
Bst2	5.56	5.20E-12	2.30E-09
Tbx2	5.54	9.25E-05	5.82E-03
Slit3	5.52	2.76E-12	1.36E-09
Fosb	5.46	2.76E-09	5.86E-07
Sp100	5.38	2.50E-07	3.36E-05
Rtp4	5.38	1.31E-10	3.80E-08
Irgm2	5.37	7.64E-09	1.43E-06
Spry1	5.37	2.34E-11	8.18E-09
Thbs1	5.32	1.83E-05	1.46E-03
Cmpk2	5.28	7.54E-08	1.13E-05
Ddx60	5.26	8.58E-08	1.26E-05
Lama4	5.26	4.46E-12	2.01E-09
Dmd	5.17	1.26E-09	2.85E-07
Nsg1	5.11	2.84E-09	5.97E-07
Ifi27	5.09	2.14E-11	7.63E-09
Adam23	5.08	6.18E-12	2.63E-09
Nbl1	5.03	2.12E-08	3.57E-06
Rora	5.02	4.43E-08	6.81E-06
Lgals3bp	5.00	4.83E-11	1.54E-08
Hoxb8	4.97	1.91E-03	6.64E-02
Oas1b	4.97	2.01E-06	2.16E-04
Lrrc17	4.93	1.15E-10	3.41E-08
Apol9a	4.91	1.01E-07	1.44E-05
Sp110	4.91	3.64E-08	5.80E-06
Clec14a	4.89	1.71E-09	3.80E-07
Parp10	4.86	6.91E-04	2.95E-02
Dact1	4.84	1.88E-08	3.24E-06

Grb14	4.79	1.01E-10	3.04E-08
Xaf1	4.70	1.17E-08	2.08E-06
Peg3	4.69	1.25E-10	3.68E-08
Sorbs1	4.64	2.61E-10	6.91E-08
Ddx4	4.60	4.93E-04	2.21E-02
Cfh	4.59	2.92E-06	2.96E-04
Pi15	4.58	9.61E-08	1.39E-05
Fzd4	4.52	7.66E-09	1.43E-06
Dpep1	4.41	3.14E-08	5.16E-06
Cacna1g	4.40	4.13E-09	8.30E-07
Alpl	4.33	2.48E-03	8.10E-02
Oasl2	4.31	1.79E-08	3.10E-06
Apol6	4.31	3.67E-04	1.77E-02
Kcnc2	4.27	9.88E-04	3.85E-02
Cyp1b1	4.26	7.37E-10	1.78E-07
Stat1	4.22	1.22E-09	2.78E-07
Cdo1	4.22	2.23E-06	2.36E-04
Nnmt	4.21	1.01E-06	1.19E-04
Cck	4.15	2.72E-03	8.72E-02
P2rx3	4.11	2.21E-07	3.01E-05
Nod1	4.03	9.81E-09	1.79E-06
Apol10b	4.01	8.86E-06	7.87E-04
Tnfrsf14	3.94	1.57E-04	8.86E-03
Adamts1	3.94	7.63E-09	1.43E-06
Mir143hg	3.89	1.01E-04	6.22E-03
Gbp7	3.87	6.65E-07	8.25E-05
Ifit3	3.79	7.75E-06	7.04E-04
Fcgrt	3.79	7.01E-04	2.97E-02
Zbp1	3.78	4.82E-04	2.19E-02

Col12a1	3.75	1.01E-08	1.82E-06
Limch1	3.74	3.63E-04	1.76E-02
Apol9b	3.73	4.21E-05	2.98E-03
H2-Q4	3.73	6.46E-07	8.05E-05
Trim34a	3.69	8.97E-04	3.59E-02
Fbln5	3.68	1.41E-08	2.47E-06
Svep1	3.65	4.29E-08	6.70E-06
Rabl6	3.65	7.43E-08	1.12E-05
Parp14	3.63	1.01E-07	1.44E-05
Ppp1r3b	3.63	6.94E-04	2.95E-02
Bmper	3.61	1.51E-07	2.12E-05
Egr1	3.59	8.98E-08	1.31E-05
Slc10a6	3.54	8.52E-04	3.44E-02
Rab40b	3.43	7.93E-04	3.28E-02
Kazald1	3.43	5.29E-04	2.36E-02
Grpr	3.43	2.45E-03	8.05E-02
C130026I21Rik	3.42	2.30E-05	1.76E-03
Rasl12	3.40	5.71E-05	3.86E-03
Vldlr	3.39	3.97E-07	5.07E-05
Ror2	3.38	6.26E-05	4.15E-03
Gm10638	3.37	3.18E-03	9.87E-02
Arhgap33	3.36	6.11E-04	2.65E-02
Gab2	3.34	1.57E-05	1.27E-03
Lpl	3.34	1.90E-07	2.66E-05
Atoh8	3.33	1.59E-06	1.82E-04
Sh2d3c	3.31	1.06E-04	6.50E-03
Fat4	3.30	3.51E-07	4.56E-05
Ifi47	3.28	2.20E-04	1.17E-02
Lifr	3.27	3.60E-07	4.64E-05

Cdkn2d	3.27	2.35E-03	7.77E-02
Hoxc13	3.26	4.77E-05	3.31E-03
Stat2	3.26	1.09E-06	1.28E-04
Ksr1	3.26	2.03E-06	2.17E-04
Ednra	3.23	3.32E-07	4.34E-05
Slc43a1	3.22	2.65E-04	1.37E-02
Kifc2	3.19	2.66E-03	8.56E-02
Boc	3.19	1.21E-06	1.40E-04
Nlrc5	3.18	1.30E-04	7.63E-03
Gpc4	3.15	4.99E-07	6.30E-05
Ifi35	3.13	4.39E-06	4.23E-04
Il17ra	3.13	3.20E-03	9.88E-02
Acss2	3.11	7.05E-06	6.45E-04
Mxd1	3.10	2.02E-05	1.59E-03
Ier2	3.06	8.79E-04	3.53E-02
Grem2	3.04	3.41E-06	3.38E-04
Zfp503	3.04	2.17E-04	1.16E-02
Zeb2	3.02	3.40E-06	3.38E-04
H2-T10	3.02	2.86E-05	2.09E-03
Hs6st2	3.02	6.68E-06	6.17E-04
Egr2	3.02	5.16E-05	3.54E-03
P4ha3	3.01	2.91E-05	2.12E-03
Nr4a1	3.01	4.59E-06	4.36E-04
Irf9	3.00	3.45E-06	3.40E-04
Adcyap1r1	3.00	1.93E-04	1.07E-02
S1pr3	2.99	2.74E-06	2.82E-04
Trim21	2.96	1.19E-04	7.15E-03
Usp2	2.96	5.43E-05	3.71E-03
Zfp36	2.94	8.61E-06	7.72E-04

Vstm2a	2.93	4.37E-05	3.06E-03
Pdgfrb	2.92	2.75E-03	8.81E-02
Pmm1	2.92	1.03E-05	8.96E-04
Gm7609	2.91	1.32E-04	7.69E-03
Arrdc4	2.91	1.33E-05	1.10E-03
Ptn	2.90	5.38E-06	5.05E-04
Tle2	2.89	2.59E-05	1.93E-03
Vdr	2.88	6.64E-06	6.16E-04
Serpine2	2.87	1.34E-05	1.11E-03
H19	2.87	1.17E-04	7.07E-03
5430417L22Rik	2.85	3.55E-05	2.55E-03
Adamts2	2.85	1.80E-05	1.43E-03
Pnp2	2.75	8.03E-04	3.29E-02
Emc9	2.74	1.78E-03	6.21E-02
Thbd	2.74	2.81E-04	1.42E-02
Zeb2os	2.73	1.21E-03	4.55E-02
Trp63	2.73	1.52E-04	8.59E-03
Gdpd2	2.72	1.01E-03	3.91E-02
Rnf149	2.71	4.90E-05	3.39E-03
Ifit1	2.70	2.19E-05	1.71E-03
Pde1a	2.68	5.29E-04	2.36E-02
Parp9	2.68	2.41E-05	1.83E-03
Ebf1	2.67	2.13E-05	1.67E-03
Rnf144a	2.66	1.40E-04	8.03E-03
C1s	2.65	8.22E-05	5.27E-03
Pid1	2.63	1.97E-04	1.08E-02
Smim1	2.63	2.78E-04	1.41E-02
Ehd3	2.61	1.15E-04	6.96E-03
Fzd7	2.60	1.42E-04	8.14E-03

H2-DMa	2.60	1.62E-03	5.75E-02
Nr2f1	2.60	9.82E-05	6.14E-03
Adamts12	2.59	7.67E-05	4.98E-03
Pdzrn3	2.59	9.07E-05	5.72E-03
Gpatch11	2.59	1.95E-04	1.08E-02
Polr3gl	2.58	7.23E-04	3.03E-02
Zcwpw1	2.57	2.05E-04	1.11E-02
Cish	2.57	9.50E-04	3.73E-02
Rtn2	2.56	2.92E-03	9.24E-02
Reck	2.56	2.93E-04	1.48E-02
Aspn	2.56	2.24E-03	7.53E-02
Egfr	2.55	4.35E-05	3.06E-03
Izumo4	2.55	2.34E-03	7.76E-02
Setbp1	2.54	5.55E-05	3.76E-03
Il1r1	2.52	5.80E-05	3.88E-03
BC018242	2.52	7.00E-04	2.97E-02
Prkce	2.51	2.20E-04	1.17E-02
Dhx58	2.51	3.18E-04	1.59E-02
Map3k6	2.50	3.27E-04	1.62E-02
Ppap2a	2.49	5.54E-04	2.44E-02
Tpcn2	2.48	2.76E-04	1.41E-02
Cdkn2c	2.47	1.45E-04	8.29E-03
Syt11	2.45	5.29E-04	2.36E-02
4933426M11Rik	2.45	1.87E-04	1.04E-02
Tll1	2.42	2.68E-04	1.38E-02
Ifitm3	2.41	8.23E-05	5.27E-03
Mgst1	2.41	2.17E-04	1.16E-02
Ppp1r3c	2.41	1.23E-03	4.62E-02
Pappa	2.39	1.28E-04	7.52E-03

Helz2	2.39	2.47E-04	1.29E-02
S1pr1	2.36	2.11E-04	1.14E-02
Snai1	2.36	5.69E-04	2.49E-02
Fstl1	2.35	1.96E-04	1.08E-02
Col6a2	2.35	6.72E-04	2.87E-02
Ppp1r15a	2.35	2.12E-03	7.24E-02
Osmr	2.35	2.14E-03	7.28E-02
Stk40	2.33	1.24E-03	4.62E-02
Nfia	2.32	2.33E-04	1.24E-02
Olfml2b	2.31	4.11E-04	1.94E-02
LOC547349	2.31	9.16E-04	3.65E-02
Col6a1	2.29	8.19E-04	3.33E-02
Fgf10	2.28	6.63E-04	2.84E-02
Il6st	2.28	2.42E-04	1.27E-02
Trim12c	2.28	7.06E-04	2.98E-02
Adc	2.26	2.22E-03	7.51E-02
Stat3	2.25	4.72E-04	2.15E-02
Lamc1	2.24	7.11E-04	2.99E-02
Pde4b	2.23	3.74E-04	1.80E-02
Per1	2.23	1.51E-03	5.41E-02
Kif26b	2.22	1.07E-03	4.10E-02
Foxs1	2.21	1.59E-03	5.63E-02
Agrn	2.21	1.38E-03	5.03E-02
Arhgef25	2.20	7.65E-04	3.18E-02
Id3	2.20	3.05E-03	9.58E-02
Sdc1	2.19	1.31E-03	4.85E-02
Ptgfr	2.19	4.58E-04	2.10E-02
Tob2	2.18	1.59E-03	5.63E-02
Mnda	2.18	2.59E-03	8.38E-02

H2-L	2.16	2.03E-03	6.99E-02
Gda	2.15	3.06E-03	9.58E-02
C1ra	2.14	1.29E-03	4.79E-02
Tef	2.14	1.76E-03	6.14E-02
Cebpb	2.14	1.57E-03	5.57E-02
Sash1	2.13	1.72E-03	6.05E-02
Ppap2b	2.13	1.01E-03	3.90E-02
Dram1	2.12	1.38E-03	5.03E-02
Dtx3l	2.12	1.19E-03	4.50E-02
Dlc1	2.11	1.11E-03	4.23E-02
Arhgap20	2.10	8.84E-04	3.55E-02
Nfkbiz	2.08	2.24E-03	7.54E-02
Mmp19	2.05	2.30E-03	7.67E-02
Lgals9	2.03	2.82E-03	8.97E-02
Sepp1	2.02	9.90E-04	3.85E-02
Gcnt2	1.96	3.24E-03	9.98E-02
Ddx58	1.96	1.78E-03	6.22E-02
Arhgef5	0.49	2.29E-03	7.66E-02
Ier3	0.49	3.22E-03	9.93E-02
Adamts7	0.49	1.43E-03	5.18E-02
Dock9	0.48	2.22E-03	7.51E-02
Ercc1	0.48	1.44E-03	5.18E-02
Adam8	0.47	1.26E-03	4.71E-02
Gprc5a	0.47	2.03E-03	6.99E-02
Apcdd1	0.46	6.11E-04	2.65E-02
Ccbe1	0.45	1.25E-03	4.66E-02
Rbpj	0.44	9.48E-04	3.73E-02
Etv4	0.44	4.65E-04	2.13E-02
Jup	0.44	2.80E-04	1.42E-02

Rbp1	0.44	6.29E-04	2.71E-02
Angptl2	0.44	1.36E-04	7.82E-03
Ttc26	0.43	2.33E-03	7.74E-02
Clcn5	0.43	7.74E-04	3.22E-02
Pdp1	0.42	1.46E-03	5.24E-02
Nfatc2	0.42	3.18E-03	9.87E-02
Syne2	0.42	1.33E-04	7.69E-03
Ccrn4l	0.41	4.24E-04	1.99E-02
Pard6b	0.41	1.21E-03	4.56E-02
Pcgf5	0.41	9.30E-04	3.69E-02
Dpt	0.41	2.52E-04	1.31E-02
Rcor2	0.40	1.01E-04	6.26E-03
Pik3cb	0.40	3.25E-04	1.61E-02
Atp6v0a4	0.40	2.82E-03	8.97E-02
Erc2	0.40	3.20E-03	9.88E-02
Slc25a13	0.40	2.72E-04	1.40E-02
Dusp4	0.40	6.66E-05	4.40E-03
Ptpre	0.39	9.91E-04	3.85E-02
Lpar2	0.39	1.08E-03	4.15E-02
Mpp6	0.39	2.46E-03	8.06E-02
Il17rd	0.39	1.64E-04	9.20E-03
Olfm1	0.38	2.29E-03	7.66E-02
5730507C01Rik	0.38	2.18E-03	7.39E-02
5430435G22Rik	0.38	1.60E-04	9.00E-03
Fam185a	0.38	6.29E-04	2.71E-02
Nfatc1	0.38	5.87E-05	3.92E-03
Tgfbr2	0.38	2.28E-05	1.75E-03
Lipg	0.37	2.81E-03	8.97E-02
Elmo1	0.37	1.73E-03	6.09E-02

Serpinb9b	0.37	3.82E-04	1.82E-02
Loxl4	0.36	1.27E-05	1.06E-03
Plat	0.36	2.42E-05	1.83E-03
Afap1l2	0.36	4.38E-04	2.03E-02
Igfbp4	0.36	1.85E-06	2.03E-04
Vav3	0.36	9.98E-05	6.19E-03
Pdpn	0.35	2.99E-03	9.42E-02
Ralgps2	0.35	1.32E-04	7.69E-03
Csf1r	0.35	1.32E-03	4.86E-02
Enpp5	0.35	4.46E-04	2.05E-02
Ptpn6	0.35	4.31E-04	2.01E-02
Cgref1	0.34	9.13E-04	3.64E-02
Adamts14	0.34	1.97E-06	2.13E-04
Chml	0.34	1.08E-04	6.61E-03
Met	0.34	5.33E-05	3.65E-03
Tanc2	0.33	9.63E-06	8.46E-04
1700088E04Rik	0.33	2.09E-03	7.14E-02
Parp16	0.33	3.06E-03	9.58E-02
Gas7	0.33	3.03E-06	3.06E-04
Dyrk3	0.32	1.07E-05	9.21E-04
Pik3r5	0.32	1.67E-05	1.34E-03
Dock11	0.32	8.01E-04	3.29E-02
Wnt6	0.31	1.18E-05	1.00E-03
Strbp	0.31	1.33E-05	1.10E-03
Cdkl2	0.31	3.24E-04	1.61E-02
Prtg	0.30	4.77E-05	3.31E-03
Arhgap22	0.30	3.06E-07	4.05E-05
Timp3	0.30	1.66E-06	1.89E-04
Ndrg2	0.29	1.77E-03	6.19E-02

Fmn1	0.29	2.11E-04	1.14E-02
Lphn3	0.29	2.89E-03	9.17E-02
Sgms2	0.29	7.70E-06	7.02E-04
Ctnnal1	0.28	1.97E-07	2.73E-05
Hspa4l	0.28	4.84E-07	6.14E-05
Prl2c4	0.27	1.33E-04	7.69E-03
Prl2c3	0.27	1.33E-04	7.69E-03
Flrt3	0.27	2.43E-06	2.54E-04
Prl2c2	0.26	4.79E-04	2.18E-02
Slc22a23	0.26	2.09E-07	2.87E-05
Mmp3	0.26	2.89E-07	3.85E-05
Lgals7	0.26	2.66E-03	8.56E-02
Bnc1	0.25	4.14E-08	6.51E-06
Pctp	0.24	2.53E-04	1.32E-02
Spon2	0.24	2.94E-09	6.13E-07
Stx3	0.24	3.34E-06	3.34E-04
Cytip	0.23	1.99E-06	2.15E-04
Ccdc41os1	0.22	1.14E-03	4.35E-02
Ngef	0.22	4.32E-04	2.01E-02
Acsbg1	0.22	3.44E-09	6.98E-07
St6gal1	0.22	4.19E-09	8.34E-07
Rhou	0.22	5.11E-10	1.27E-07
Macc1	0.22	1.36E-03	4.99E-02
Pkp3	0.22	2.72E-05	2.00E-03
Klk10	0.21	4.90E-04	2.21E-02
Lrrc8d	0.21	2.67E-09	5.73E-07
Atp8b4	0.21	2.63E-06	2.73E-04
Gm12603	0.20	1.24E-04	7.32E-03
Nipal1	0.20	8.54E-04	3.45E-02

Snora44	0.20	4.41E-04	2.05E-02
Trpv4	0.20	4.21E-06	4.11E-04
Ccl17	0.20	8.13E-04	3.32E-02
Sema3f	0.19	6.59E-11	2.07E-08
Prrg4	0.19	1.98E-07	2.73E-05
Lamb3	0.19	1.48E-04	8.42E-03
Il34	0.19	6.19E-06	5.76E-04
Cd300lb	0.18	5.87E-06	5.49E-04
Chrnb1	0.18	1.11E-05	9.56E-04
Pdgfb	0.18	2.30E-06	2.42E-04
Nlrc3	0.18	9.34E-06	8.27E-04
Il18rap	0.18	1.50E-11	5.72E-09
Ntrk3	0.18	1.79E-06	2.01E-04
Etl4	0.18	2.09E-08	3.54E-06
Fyb	0.17	2.64E-03	8.52E-02
Ptprb	0.17	1.32E-03	4.86E-02
Sema7a	0.17	1.70E-11	6.35E-09
Clec1a	0.17	1.18E-03	4.48E-02
Itga3	0.17	6.54E-13	3.91E-10
Creb5	0.16	1.08E-04	6.60E-03
Plac1	0.16	1.47E-04	8.40E-03
Gdpd5	0.16	2.08E-10	5.79E-08
Card10	0.16	4.27E-12	1.97E-09
Accsl	0.16	4.09E-05	2.90E-03
Ctsw	0.16	2.37E-04	1.25E-02
Eml5	0.15	9.93E-13	5.35E-10
Hcn4	0.15	5.59E-04	2.45E-02
Sema5a	0.15	1.07E-13	7.04E-11
Rap1gap	0.15	2.51E-07	3.37E-05

Cln3	0.14	4.88E-04	2.21E-02
Atg9b	0.14	1.05E-05	9.09E-04
Xpnpep2	0.13	1.64E-12	8.46E-10
Dock8	0.13	3.34E-08	5.41E-06
Myh1	0.12	3.53E-04	1.72E-02
Cd33	0.12	2.39E-05	1.82E-03
Zfp709	0.12	7.46E-04	3.12E-02
S100a16	0.12	5.60E-09	1.07E-06
Ano1	0.12	9.48E-09	1.74E-06
Sorcs2	0.12	6.32E-17	8.57E-14
Adcy7	0.12	1.67E-16	2.14E-13
Htra1	0.12	6.97E-11	2.16E-08
Cgn	0.12	3.10E-12	1.50E-09
Pla2g7	0.12	1.09E-15	1.17E-12
Gna14	0.11	5.45E-04	2.40E-02
AU021092	0.11	3.03E-11	1.01E-08
Itgb7	0.11	1.14E-15	1.17E-12
Pkp1	0.11	8.12E-05	5.23E-03
Tenm4	0.11	3.66E-19	7.94E-16
Glrp1	0.10	2.29E-03	7.66E-02
Phex	0.10	1.77E-10	4.99E-08
Krt8	0.08	9.82E-07	1.16E-04
Cyth4	0.08	1.01E-03	3.90E-02
Podxl	0.07	1.13E-04	6.86E-03
Ngfr	0.07	1.28E-05	1.07E-03
Dynap	0.06	3.70E-14	2.68E-11
Inpp4b	0.05	4.42E-05	3.08E-03
Lgr6	0.04	4.71E-20	1.28E-16
Blnk	0.04	3.34E-06	3.34E-04

Akr1c18	0.04	1.48E-19	3.57E-16
Slc14a1	0.03	9.97E-11	3.04E-08
Plet1	0.03	1.92E-06	2.09E-04
Sh2d1b1	0.01	8.36E-12	3.36E-09

#. Fold change is the ratio of expression of genes in P versus C cells at -2 day.

$. Benjamini-Hochberg adjusted p-value.

Supplementary Table S1

The list of genes similarly affected by Prep1 DR at day -2 and by induction of adipogenic differentiation control cells at day 1.

G.O. Category	P-value	P adj#	Gene symbols
fat cell differentiation (GO:0045444)	8.45E-04	3.06E-02	CEBPA; CEBPB; EGR2; PID1; FABP4; LAMA4; RARRES2; PSMB8; FGF10
response to cAMP (GO:0051591)	2.59E-05	3.40E-03	EGR1; THBD; EGR2; IGFBP5; STAT1; PDE3A;FOSB; MMP19; SDC1; FOS; CDO1
response to glucocorticoid (GO:0051384)	5.19E-04	2.33E-02	CEBPA; PAPPA; SDC1; FOSB; ALPL; ADM; SLIT3; AVPR1A; FOS; CDO1; EGFR
single organism cell adhesion (GO:0098602)	2.33E-07	5.09E-04	CYFIP2; LAMA5; CDSN; JUP; PIK3CB; FNDC3A; SLC7A11; MERTK; EFNA1; PDPN; PKP1; ITGA6; PTPN6; ITGB7; ADAM8; PKP3; CD34

#. Benjamini-Hochberg adjusted p-value.

Supplementary Table S2

Statistically significant enrichments for the gene ontology categories highlighted in Fig. 2D. Genes in these categories are shown.

-2 days	C exc[#]	Common* (C and P)	P exc
GO 0045444<br>fat cell differentiation	1	9	18
GO 0045598<br>regulation of fat cell differentiation	0	5	13
GO:0045600<br>positive regulation of fat cell differentiation	0	2	7
GO 0045599<br>negative regulation of fat cell differentiation	0	3	5

4 hours	C exc	Common (C and P)	P exc
GO 0045444<br>fat cell differentiation	0	56	42
GO 0045598<br>regulation of fat cell differentiation	0	58	22
GO:0045600<br>positive regulation of fat cell differentiation	0	26	11
GO 0045599<br>negative regulation of fat cell differentiation	0	23	8
GO 0050873<br>brown fat cell differentiation	0	21	9
GO 0050872<br>white fat cell differentiation	0	9	2

#. exclusive genes. Peak-associated genes present only in P (or C) cells

*. common genes. Peak-associated genes present in both P and C cells.

Supplementary Table S3

Distribution of the enriched G.O. categories and the corresponding number of genes in P and C cells at -2 day and 4 hours after induction.
